# Monitoring decellularization via absorbance spectroscopy during the derivation of extracellular matrix scaffolds

**DOI:** 10.1088/1748-605X/ac361f

**Published:** 2021-11-26

**Authors:** Camilo Mora-Navarro, Mario E Garcia, Prottasha Sarker, Emily W Ozpinar, Jeffrey R Enders, Saad Khan, Ryan C Branski, Donald O Freytes

**Affiliations:** 1 Joint Department of Biomedical Engineering, North Carolina State and University of North Carolina-Chapel Hill, Raleigh, NC, United States of America; 2 Comparative Medicine Institute, North Carolina State University, Raleigh, NC, United States of America; 3 Department of Chemical and Biomolecular Engineering, North Carolina State University, Raleigh, NC 27695, United States of America; 4 Molecular Education, Technology and Research Innovation Center, North Carolina State University, Raleigh, NC, United States of America; 5 Department of Biological Sciences, North Carolina State University, Raleigh, NC, United States of America; 6 Departments of Rehabilitation Medicine and Otolaryngology-Head and Neck Surgery, NYU Grossman School of Medicine, New York, NY, United States of America; 7 Department of Chemical Engineering, University of Puerto Rico, Mayaguez, PR, United States of America

**Keywords:** extracellular matrix, manufacturing, decellularization, monitoring, vocal fold

## Abstract

Extracellular matrix (ECM) is a complex structure composed of bioactive molecules representative of the local tissue microenvironment. Decellularized ECM biomaterials harness these biomolecules for regenerative medicine applications. One potential therapeutic application is the use of vocal fold (VF) specific ECM to restore the VFs after injury. ECM scaffolds are derived through a process of decellularization, which aims to remove unwanted immunogenic biomolecules (e.g. DNA) while preserving the composition of the ECM. The effectiveness of the decellularization is typically assessed at the end by quantifying ECM attributes such as final dsDNA content. However, batch-to-batch variability in ECM manufacturing remains a significant challenge for the standardization, cost-effectiveness, and scale-up process. The limited number of tools available for in-process control heavily restricts the uncovering of the correlations between decellularization process parameters and ECM attributes. In this study, we developed a technique applicable to both the classical batch method and semi-continuous decellularization systems to trace the decellularization of two laryngeal tissues in real-time. We hypothesize that monitoring the bioreactor’s effluent absorbance at 260 nm as a function of time will provide a representative DNA release profile from the tissue and thus allow for process optimization. The DNA release profiles were obtained for laryngeal tissues and were successfully used to optimize the derivation of VF lamina propria-ECM (auVF-ECM) hydrogels. This hydrogel had comparable rheological properties to commonly used biomaterials to treat VF injuries. Also, the auVF-ECM hydrogel promoted the down-regulation of CCR7 by THP-1 macrophages upon lipopolysaccharide stimulation *in vitro* suggesting some anti-inflammatory properties. The results show that absorbance profiles are a good representation of DNA removal during the decellularization process thus providing an important tool to optimize future protocols.

## 1. Introduction

Decellularized extracellular matrix (ECM) biomaterials are attractive due to their potential use across clinical, research, and 3D printing applications as a more physiologically relevant substrate. They also possess favorable wound healing properties [1–3]. ECM is mainly composed of structural proteins and other bioactive molecules that can guide cellular responses providing tissue-specific biochemical and biomechanical cues [4–6]. ECM-based biomaterials take advantage of the biomolecules deposited by tissue-resident cells to form tissue-specific, complex 3D structures necessary for tissue function as well as the signaling necessary to coordinate the response to injury [7]. These properties make the ECM an ideal but complex scaffold with broad regenerative medicine applications. All these properties rely on the proper decellularization method that can retain the biomolecules responsible for the host tissue response while removing unwanted components. Therefore, understanding and controlling the decellularization process is vital to their large scale manufacturability and clinical success.

ECM based biomaterials are most often derived via tissue decellularization, typically from porcine organs/tissues, given their ubiquitous availability. Tissue decellularization commonly involves sequential treatments to remove unwanted immunogenic factors with the potential of driving host rejection (e.g. DNA) while preserving the complex ECM architecture and composition [8]. Ideally, once an adequate decellularization protocol is identified (i.e. reagent sequence, exposure time, agitation, etc), the method is then consistently executed in order to minimize variability of the final ECM attributes. However, significant variation in final DNA content in the ECM products (batch to batch variability) remains a challenge in the field and can result in the rejection of entire batches, negatively impacting the cost-efficiency of the process [9].

Biofabrication of ECM-based biomaterials and the final quality attributes largely depend on the decellularization process. Established decellularization workflows rely on evaluating decellularization effectiveness by measuring variables such as residual nuclei or DNA content [10, 11]. This kind of endpoint evaluation workflow does not offer a practical path towards identifying, detecting, and adjusting real-time process parameters such as reagent exposure times and reagent sequence. Arguably, translation and upscaling of ECM manufacturing could be challenging due to limited understanding of the correlation between decellularization parameters and the final ECM quality. This quality control approach restricts the identification of process parameters that can be used to increase efficiency for the classical batch decellularization (sheet method, sh) and the semi-batch automated method (au) [12]. Badileanu *et al* developed a semi-batch decellularization process for soft tissues with automated reagent dosing (inputs and effluent) to address the user-driven variability during the process [13]. The significance of the automated method is primarily related to semi-continuous inline filtration to enable time-programed dosing pumps to deliver reagents and remove waste. The automated system is an initial step to standardize decellularization for ECM-manufacturing and to implement inline monitoring to facilitate process control and optimization in real-time. However, to the best of our knowledge, in-line DNA removal tracing for manual or automated decellularization has not been performed. Ultimately, a method for the in-line monitoring process is critical to understand the decellularization dynamics, control the batch-to-batch variability, and improve the efficiency of the ECM manufacturing process.

Absorbance spectroscopy is widely available and commonly employed to monitor nucleic acids in aqueous solutions (i.e. dsDNA, ssDNA, RNA, etc) [14]. We hypothesize that monitoring the bioreactors effluent’s absorbance at 260 nm as a function of reagent exposure time will provide a representative DNA release profile (potential monitoring curves) from the tissue into the liquid phase (i.e. reactors effluent). This profile can then be correlated to decellularization parameters. This information will be used to determine the overall status of decellularization and improve ECM derivation.

In the current study, we investigated the use of absorbance curves to understand the dynamics of the decellularization process and generate decellularization profiles for two laryngeal soft tissues: the vocal fold lamina propria (VFLP) and supraglottic (SG) tissues. Both tissues could have profound clinical applications for patients suffering from vocal fold (VF) injuries for which a truly regenerative treatment remains elusive. Large scale production of such materials will be needed for a viable commercial product. Therefore, our goal was to measure the effect of reagents and exposure times at different scales during the decellularization process and to use such information to optimize the development of an injectable and cytocompatible hydrogel for VF applications. These profile curves are the first of their kind and have the potential to drive future studies to optimize decellularization protocols for either classical or automated workflows. Although targeted for VF-specific ECM, this technique allows for the collection of real-time information to be used as an input to a control system for manufacturing ECM products at a large scale with full quality compliance.

## 2. Materials and methods

### 2.1. Tissues

Porcine larynges (Nahunta Pork Outlet) were dissected, and the mucosa from the true VF and SG were removed. The tissues were then frozen at −20 °C for at least 24 h.

### 2.2. Decellularization and monitoring curves

#### 2.2.1. Classical sheet method (sh-method) and absorbance spectroscopy recording data

Porcine VFLP and SG decellularization was performed as previously described [15]. SGs were divided into pieces closer in size to VFLP. The raw tissue was centrifuge at 3000 RPM for 30 s and at least three independent batches from 1 g each were weight. Tissues were treated with 25 ml of the following solutions under constant agitation on an orbital shaker: three times with 1× DPBS for 15 min each, 4% w/v sodium deoxycholate (NaDeox) (Sigma-Aldrich) for 2 h, 1× DPBS for 15 min, deoxyribonuclease (DNase) I (Sigma-Aldrich) at 273 Kunitz/mL in PBS pH 7.4 supplemented with 2.5 mM Mg^2+^ and 0.1 mM Ca^2+^ for 2 h, 1× DPBS for 15 min, 0.1% v/v peracetic acid (Sigma-Aldrich) in 4% v/v ethanol solution for 30 min, 1× DPBS for 15 min. See table S1, part A for a summary of decellularization steps. During each decellularization stage (i.e. Wash, Detergent, and Nuclease), samples of 0.2 ml were taken from the system to measure absorbance at 260 nm using a Nanodrop 2000 (ThermoFisher) shortly after sample collection.

#### 2.2.2. Semi-batch automated method (au-method) and absorbance spectroscopy recording data

Porcine VFLP and SG were separately ground after freezing overnight using a Ninja Blender (Ninja®). The minced initial native tissues were centrifuged at 3000 RPM to remove the blood excess and adjusted to yield at least three independent batches per condition (0.5 g, 1 g, and 2 g) to be decellularized as previously described [13]. Micronized tissues were then placed in a custom-made bioreactor (as described by Badileano *et al*) and treated with the 25 ml of the solutions listed in table S1, part B under constant agitation. An additional 3 mm silicone tubing was inserted near the waste outlet for sample collection. Decellularized auVFLP-ECM or auSG-ECM scaffolds were lyophilized. Three exposure times were tested, but the reagent sequence and concentrations were preserved as described in table S1, part (i, ii, & iii). At least three independent decellularization per tissue per condition were performed (*n* = 3). During each decellularization stage, 0.2 ml samples were taken from the solution via sampling line at the corresponding exposure time to measure the absorbance at 260 nm; all measurements were conducted shortly after the sample collection.

### 2.3. Decellularized ECM characterization

#### 2.3.1. Double strand DNA (dsDNA) quantification

Native and decellularized ECM scaffolds were lyophilized overnight. Approximately 3 mg per sample was then digested in 20 μl (at 20 mg ml^−1^) Proteinase K Solution (Qiagen) and 180 μl Buffer ATL (Qiagen) overnight at 60 °C. Digested samples were diluted and mixed thoroughly using 800 μl of TE pH 7.4 buffer (ThermoFisher Scientific). A second dilution (1:50) was prepared using the same buffer. Further dilutions were required for native samples to reach a signal within the range of the standards supplied by the kit. dsDNA quantification was performed using the QuantiFluor dsDNA System kit (Promega) according to the manufacturer’s instructions. Samples were read using an Infinite M200 Pro plate reader (Tecan).

#### 2.3.2. Histology

Tissue samples of native and decellularized auVFLP-ECM and auSG-ECM were fixed in 4% formaldehyde (Sigma-Aldrich) overnight and stored in 70% ethanol. The samples were trimmed and sectioned at 5 μm and subjected to Hematoxylin and Eosin (H&E) and Tri-Chrome staining. Staining was performed at the Histology Laboratory in the College of Veterinary Medicine at North Carolina State University.

#### 2.3.3. Proteomics

A pool of dissected porcine VFLPs and SGs were minced to produce three independent decellularized auVFLP-ECM or au-SG scaffolds. Discovery proteomics workflow was used to characterize and compare the overall protein composition of decellularized auVFLP-ECM and auSG-ECM scaffolds.

##### 2.3.3.1. Sample preparation

To generate adequate data for statistical analysis ∼10 mg each of auVFLP-ECM or au-SG was used. Samples were suspended in 1 ml of 50 mM ammonium bicarbonate (pH = 8.0) with 5% w/v sodium deoxycholate (SigmaAldrich) for digestion and homogenization. Samples were homogenized using an OMNI tissue homogenizer and a Fisher Scientific Sonic Dismembrator Model 120. Samples were then centrifuged at 10 000 x g for 10 min, and a bicinchoninic acid assay (BCA assay) was performed on the supernatant. For BCA, samples were normalized to 50 ug of protein, and filter-aided sample preparation protocol was employed to digest and clean the samples prior to mass spectrometry [16].

##### 2.3.3.2. Liquid chromatography-mass spectrometry (LC-MS/MS)

All samples were run using a Thermo Scientific Easy Nano-LC 1200 with an EASY-Spray source complexed to a ThermoFisher Scientific Orbitrap Exploris 480. A ThermoFisher Scientific Acclaim PepMap 100 trap column (C18 LC Columns, 3 μm particle size, 75 μm ID, 20 mm length) was utilized in line with an EASY-Spray analytical column (2 μm particle size, 75 μm ID, 250 mm length) at 35 °C. The mass spectrometer was run in data-dependent acquisition mode with a cycle time of 2 s. 2 μl of volume was injected for each sample, containing approximately 1 μg of total protein.

##### 2.3.3.3. Data analysis

Raw data were loaded into Proteome Discoverer (PD) 2.4.0.305 (ThermoFisher Scientific) for analysis. A label-free quantification (LFQ) workflow was used, and the data were normalized by total peptide amount using PD. For peptide searching, the protein FASTA database was downloaded via PD from SwissProt (fully annotated) and TrEMBL (unreviewed proteins) databases for Sus Scrofa (taxonomy ID = 9823). A maximum of two equal mods were used per peptide. The following post-translational modifications were accommodated in the search algorithm (modified amino acids in parentheses): oxidation (M), deamidation (N, Q). Gene Ontology (GO) functional classification analysis (protein class) was performed using the ‘Gene List Analysis’ tool from www.pantherdb.org using Sus scrofa as the organism, and plots were constructed using PRISM or BioVenn [17, 18]. Associated ECM proteins and subunits were listed according to Naba *et al* [19].

#### 2.3.4. Hydrogel formation and Rheological measurements

Decellularized classical sh-ECM samples were powder using a mortar and pestle with liquid nitrogen to yield a powder. Automated decellularized ECM samples were lyophilized and ground in a mill. The ECM powder was digested at 10 mg ECM/mL with 0.6 mg ml^−1^ of pepsin in 0.01 M HCl for 48 h on a stirring plate [20]. ECM-hydrogels were formed by adjusting the salt concentration with 10X DPBS, neutralizing the digested ECM solution with 0.1 M NaOH, and raising the volume with water to a final ECM concentration of 6 mg ml^−1^. ECM-hydrogels were formed by aliquoting into tissue culture-treated plates or any other surface and placing at 37 C for 30–60 min. Collagen type I (Col.1 h) solution obtained from bovine hides (Advanced Biomatrix) was used as a control following the manufacturers’ instructions using 10X DPBS, 0.1 N NaOH, and water to a final Col.1 h concentration of 6 mg ml^−1^. The solubilized ECM was filtered to have a final particle size of 300 μm or smaller. Aliquiotes of 0.9 ml of filtered ECM digested were then stored at −20 °C until use. Just prior to performing the rheological experiments, the ECM was thawed on ice and adjusted to a final concentratrion of 6 mg ml^−1^. The pH of the ECM was to physiological pH (7.3 ± 0.2) using 0.1 M NaOH, phenol red as indicator and 10× DPBS for balancing the salt content. The neutralization was performed for collagen type 1 in a similar manner and adjusted tto a final concentration of 6 mg ml^−1^.

Dynamic rheological properties of ECM hydrogels were quantified using a Discovery Hybrid Rheometer-3 (TA Instruments). Digested ECMs from different batches were used for each experimentation. A 40 mm sand-blasted parallel plate geometry was used to avoid wall slip. The plates were maintained at 37 °C with a sample gap of 1 mm for all our experiments. To ensure consistency, a conditioning step was performed prior to data acquisition; samples were subjected to a dynamic pre-shear at 0.1 rad s^−1^ for 10 s, followed by an equilibration of 120 s. All experiments were conducted within the linear viscoelastic regime (LVE) verified via strain sweeps between 0.1% and 100% strain at a constant frequency of 1 rad s^−1^. Two sets of experiments were performed. The first samples were loaded and elastic (G’) and viscous (G”) moduli were monitored as a function of time at a constant frequency of 1 rad s^−1^ to observe gelation. The time sweep experiment was followed by a frequency sweep to investigate gel characteristics. The initial/pristine sample temperature loaded in the rheometer was at 4 °C. During the time sweep, the temperature of the sample increased from 4 °C to 37 °C in a short period (<30 s) playing a minimal role in the gelation monitoring process. Experiments for Collagen type I hydrogel (Col.1 h), shSG-ECMh, and auSG-ECMh were run at 2.5% strain and shVFLP-ECMh and auVFLP-ECMh were conducted at 5% strain. The frequency sweep was performed between 0.1 rad s^−1^ (0.01592 Hz) to 100 rad s^−1^ (15.92 Hz). All experiments were conducted in triplicate with a relative error of ±10%.

#### 2.3.5. Cytocompatibility using monocyte culture and macrophages (Mϕs) differentiation

THP-1 monocytes (ATCC) were cultured at a concentration of 2–4 × 10^5^ cells mL^−1^ in RPMI Media 1640 GlutaMAX™ supplemented with 10% heat-inactivated fetal bovine serum, and 1% penicillin-streptomycin and subcultured at a concentration of 8 × 10^5^ cells ml^−1^. Monocytes were differentiated into macrophages (Mϕs) through the addition of 320 nM phorbol-12-myristate 13-acetate (PMA) overnight in 75 cm^2^ ultra-low attachment flasks at a concentration of 1 × 10^6^ cells mL^−1^ and subsequently washed three times with media, as previously reported [21, 22].

##### 2.3.5.1. LPS Titration

On Day 5 of Mϕs differentiation, 2.5 × 10^5^ cells/well were seeded on the hydrogels (auVFLP and Col.1 h) or low binding tissue culture plastic (TCP). Lipopolysaccharides from E. coli (Millipore Sigma) were added at 100, 50, and 0 ng ml^−1^. After 48 h, samples were lysed in the well using TRK Lysis provided by the E.Z.N.A. Total RNA Kit I (Omega Biotek) and frozen at −80 °C for at least 24 h.

##### 2.3.5.2 Real-time quantitative polymerase chain reaction (RT-qPCR)

Samples were thawed, and RNA was isolated using the E.Z.N.A. Total RNA Kit I (Omega Bio-Tek). RNA concentrations were quantified via Nanodrop, and cDNA was synthesized with the GoScript RT System (Promega) in a SimpliAmp thermocycler (Applied Biosystems). RT-qPCR was performed using SYBR Select Master Mix in a Quantstudio 3 (Applied Biosystems) using the primers in table 1. Gene expression was normalized to GAPDH using Microsoft Excel and exported to Prism 9 (GraphPad Software) for graphing and statistical analysis. Significance was calculated using two-way ANOVA and Tukey’s statistical hypothesis testing (*α* = 0.05).

### 2.4. Statistical analysis

GraphPad PRISM 9.0 software was employed for statistical analyses. All experiments were performed in triplicate, at least, unless otherwise noted. Proteomic discovery analysis was performed using ThermoFisher PD 2.4 308 with an ANOVA hypothesis test (individual proteins). Student unpaired t-test with Welch’s correction was performed for dsDNA quantification analysis. A value of *P* < 0.05 was considered significant unless otherwise noted.

## 3. Results

### 3.1. Overall approach

**Figure 1.**
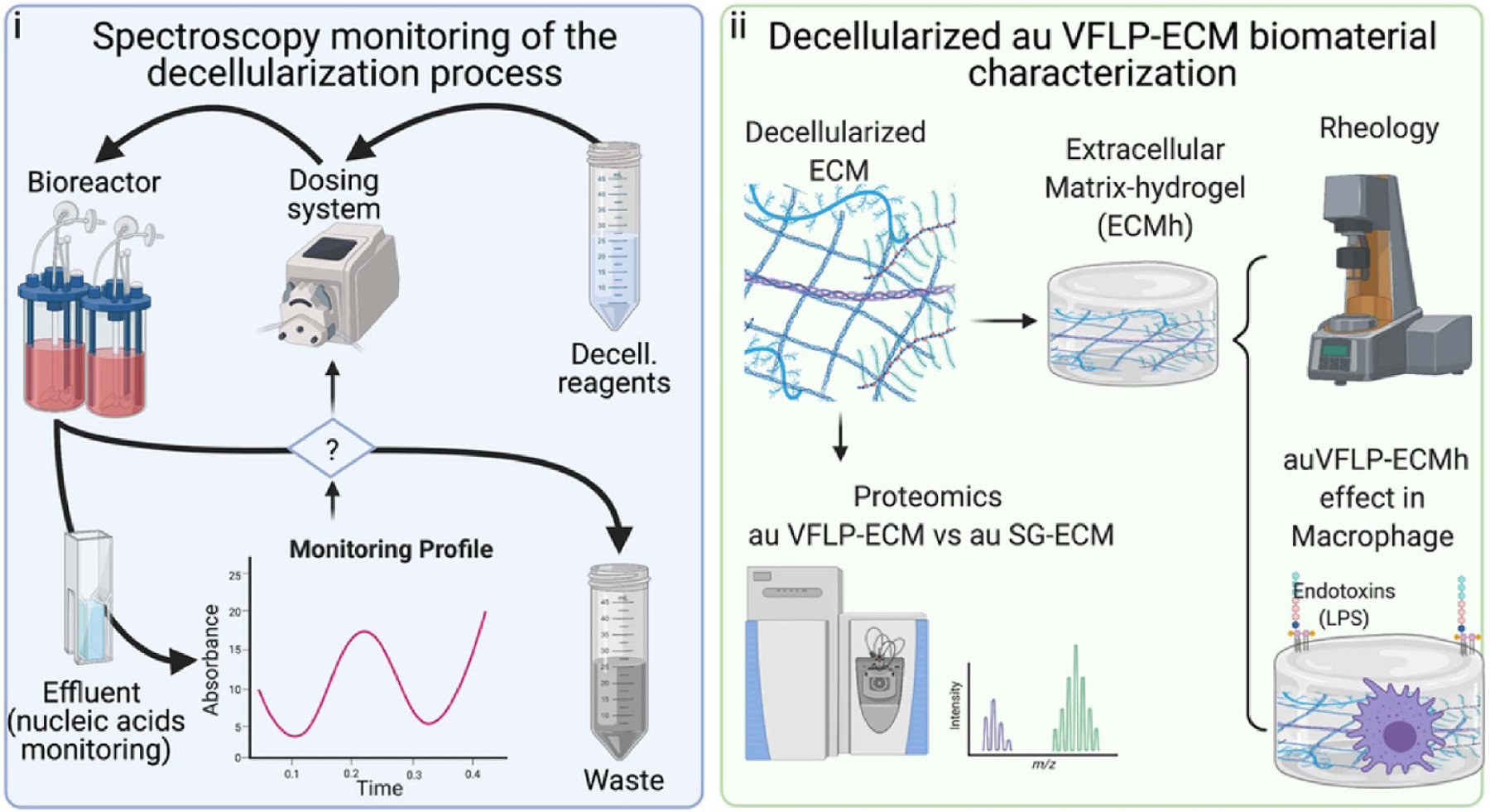
Project overview.( i) Workflow for at process determination of the absorbance at 260 nm from the bioreactor’s effluent. (ii) Derived extracellular matrix (ECM) biomaterial characterization centered on an injectable scaffold for vocal fold application.

The dynamics of tissue decellularization remain poorly controlled and characterized, making optimization of protocols a challenging task. Residual dsDNA content within decellularized ECM-biomaterials is a quality control parameter used to evaluate the effectiveness of the decellularization process [23–25]. A robust technique for tracing nucleic acid removal from the tissue would be valuable, mainly if this signal works as feedback enabling real-time monitoring to facilitate adjustments of the process’s parameters to control and optimize ECM outputs. Figure 1 illustrates our overall research approach. As shown in figure 1(i), absorbance data at 260 nm (Abs. 260 nm) from the bioreactor outputs is collected and plotted as a function of reagent exposure time. Aliquots from the bioreactor were taken during the decellularization process during either the classic batch (sheet, sh) or the semi-batch (automated, au) methods.

The dynamics of the nucleic acids (e.g. DNA) removal were assessed by plotting absorbance from the samples over time. Figure 1(ii) depicts the workflow for protein composition analysis as well as other properties from decellularized ECM derived from the au or sh-methods. As the chemical environment from the different decellularization reagents used during the process may interfere with the Abs. 260 nm measurement, we evaluated this signal using standard lambda DNA diluted into different reagent formulations commonly use during a decellularization process (figure S4 (available online at stacks.iop.org/BMM/17/015008/mmedia)). The results were linear for the conditions assayed, but the mix between NaDeox-TE buffer affected the linearity of the intensity values at lower dsDNA concentrations.

### 3.2. Monitoring profile using a classical batch decellularization approach

**Figure 2.**
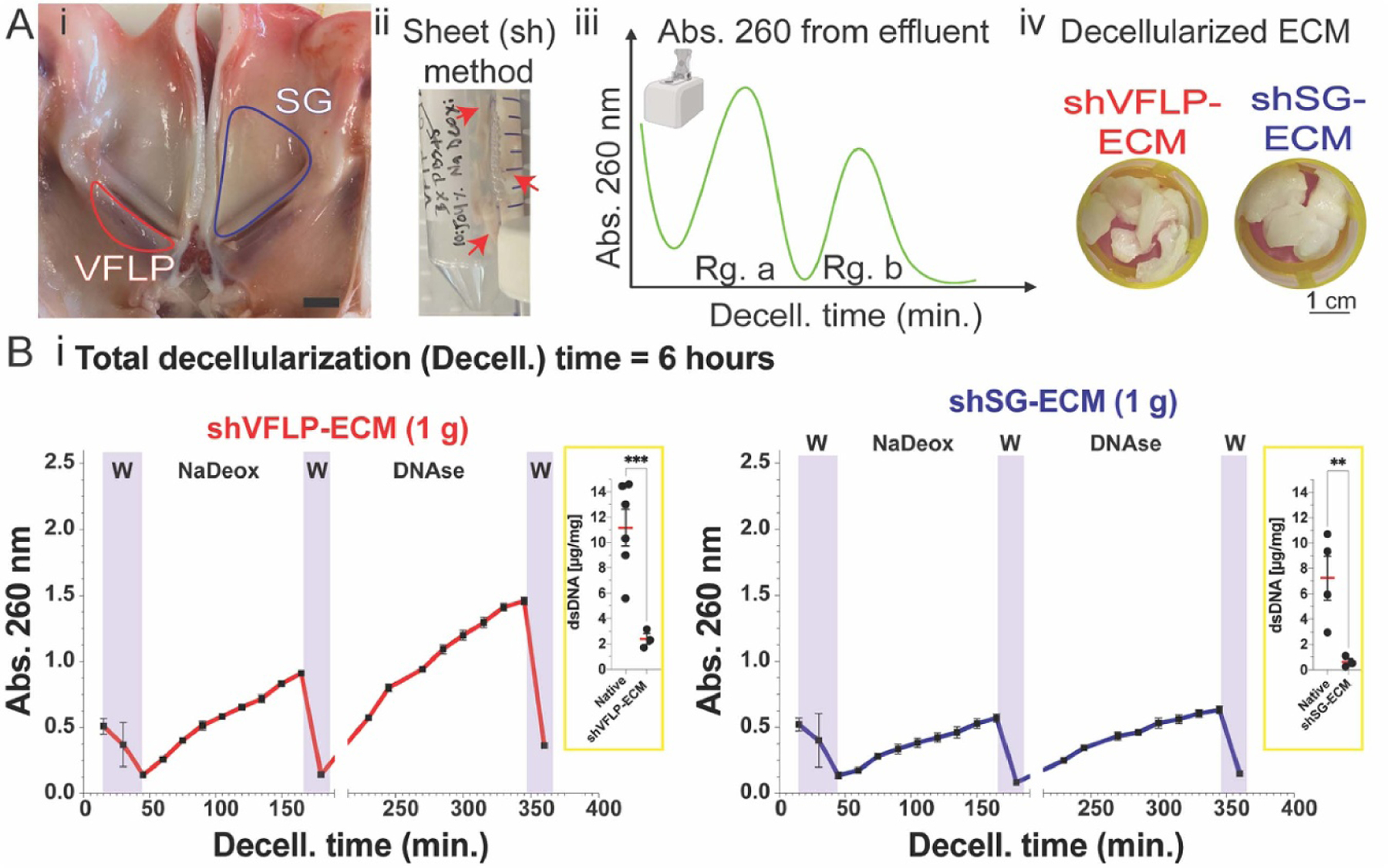
Sh-ECM production and Abs. 260 nm as a monitoring system. A (i) Open view of the porcine laryngeal area showing VFLP and SG tissue before dissection. (ii) VFLPs dissected and in the process of being decellularized using the classical sheet (sh) method. (iii) Approach to plot the absorbance intensity at 260 nm from the samples taken as a function of the decellularization time per reagent stage. (iv) Macroscopic view of the decellularized tissues after classical method. B (i) Monitoring profiles obtained for the classical decellularization method for either VFLP or SG tissue per decellularization stage. The break in the *x*-axis represents an overnight storage step (not added to the total decellularization time). The nested sub-plots display the double strand DNA quantification for the native tissue and decellularized-ECM. The error bars represent the SEM (*n* = 3), *p-values < 0.05.

VFLP and SG are proximal anatomic regions in the upper airway, as shown in figure 2(Ai). Injectable biomaterials derived from decellularized VFLP or SG are likely to maintain tissue-specific ECM composition. VFLPs and SGs were dissected from porcine airways, as described in the methods, and decellularized via the classical sh-method as shown (figure 2(Aii); see supporting table S1 for further decellularization information). Aliquots of decellularization reagents were taken from the bioreactor and the Abs. 260 nm was measured. Absorbance baseline or blank was set for each decellularization stage using each reagent at time 0 (before tissue exposure). Data were plotted as a function of exposure time to generate a curve of nucleic acid extraction from the tissue to the liquid phase or a monitoring profile from the process of decellularization. The final decellularized biomaterials, shVFLP and sh-SG, are shown in figure 2(Aiv).

The manual decellularization method (sh-method) for either VFLP or SG, used gentle rocker agitation and approximately 1± 0.15 g of tissue (5–6 pieces between 1 and 2 cm each) in a 6 h long protocol. The monitoring profiles obtained are shown in figure 2(Bi). Four regions of interest were noted in the plots: washes (W), detergent (NaDeox), O/N (overnight), and nuclease (DNAse). The purple-colored W area represents the times when the tissue was subjected to washes where a decaying trend in absorbance was observed. The profile curve obtained for the NaDeox stage showed a sigmoid-like increase in the signal as a function of time. The curve had a linear-like trend between 50 and 80 min, and it did not plateau after 120 min of reagent exposure time. The break in the *x*-axis after 190 min represents a storage step (overnight not longer than 16 h); this time was not included in the total protocol duration. The storage condition allowed for the last decellularization steps to be completed sequentially in the same day (figure S1).

The curve within the DNAse region showed a similar trend to the NaDeox profile. Signal intensity increased in response to reagent exposure time in a sigmoid-like shape, but a plateau was not observed. For VFLP, absorbance during the DNAse treatment was approximately 1.5× higher than absorbance of the NaDeox stage. Increased absorbance was observed in VFLP compared to SG, which may suggest larger quantities of nucleic acid were released to the liquid phase from VFLP tissue. dsDNA content for both materials significantly decreased after decellularization compared to raw or native tissue.

### 3.3. Monitoring profile using a semi-batch decellularization approach (au)

**Figure 3.**
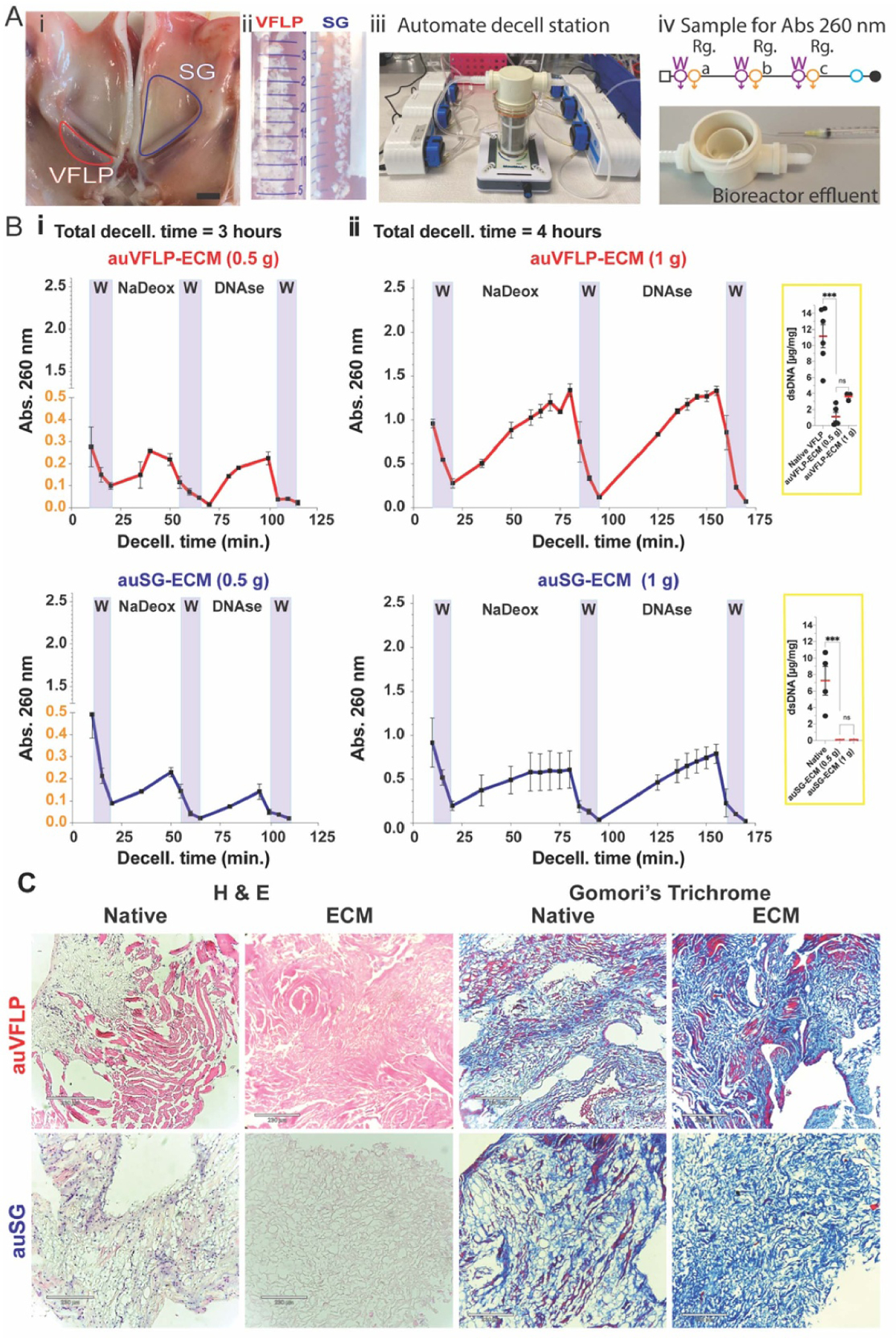
A (i) Open view of laryngeal area showing VFLP and SG tissue delimited before dissection. (ii) VFLPs and SG native tissue (raw) minced in pieces < 3 mm before being load to the semi-batch bioreactor. iii) Automated decellularization station showing the semi-batch bioreactor and the dossing system. (iv) Decellularization workflow showing the sequential decellularization stages (W= wash and reag. = reagents a, b, or c). Also, the figure shows the sample inline port used for aliquot collection. B (i) Monitoring profiles obtained for the automated decellularization method for 0.5 gr of either VFLP or SG tissue, following the protocol listed in table S1 B(i). (ii) Monitoring profile obtained for the automated decellularization method for 1 gr of either VFLP or SG tissue, following the protocol listed in table S1 B(ii). The nested sub-plots display the double strand DNA quantification for the native tissue and decellularized-ECM. C Histology staining of native tissue and automated decellularized ECM. The error bars represent the SEM (*n* = 3), * *p*-values < 0.05.

The automated (au) decellularization process used ground tissue (particle size < 3 mm) as a raw native material fed into the bioreactor, as shown in figure 3(Aii). The bioreactor had an inline vertical filter and a fluidic system controlled via automated dosing pumps (figure 3(Aiii)). Once the material was loaded into the bioreactor, dosing pumps ran the protocol by adding or removing reagents following pre-programmed exposure times, and magnetic stirrer bars homogenized the solution [13]. Aliquots from the effluent process were collected using an inline collection method (figure 3(Aiv)). Abs. 260 nm from the aliquoted samples was measured and then plotted as a function of reagent exposure time to obtain spectroscopy curves associated with decellularization. Decellularization parameters were as follow: (i) 3 h decellularization and 0.5 g of native tissue or (ii) 4 h decellularization and 1 g of native tissue (see supporting table S1).

The resulting curves are shown in figures 3(Bi) and (Bii). Within the W areas, the absorbance decreased following each wash. For NaDeox, a linear trend was observed within the first 30 min of exposure for both auVFLP and auSG. For auVFLP, the curve for DNAse treatment plateaued between 80 and 90 min, different from the DNAse profile for auSG (figure 3(Bi)).

The effect of doubling the quantity of initial tissue and extending exposure time from 30 min to 60 min for both NaDeox and DNAse is shown in figure 3(Bii). An increment in signal intensity were observed, suggesting a correlation with the initial amount of loaded tissue; this mass-dependent factor is further depicted in figure S2A. Also supporting figure S7 shows the absorbance A260/A280 ratio for the condition shown in figure 3(Bii) au-SG-ECM. The higher ratio A260/A280 in the effluents suggests a larger amounts of nucleic acids in comparison to proteins.

For the auVFLP decellularization, similar intensities were observed for both NaDeox and DNAse treatment. The NaDeox area profile shows a linear incremental trend with an earlier indication of a plateau trend between 60 and 75 min. However, in the case of DNAse and after 130 min, the curve indicates a plateau trend suggesting the system reached a potential endpoint for the treatment. The auSG decellularization profile revealed a well-defined plateau area for the NaDeox stage, but this trend was less clear than DNAse. The DNAse curve for auSG trend suggested a decay in absorbance after 130 min. Analogous to the classical sh-method, the intensity of the Abs.260 nm signal was higher for auVFLP than auSG. Native VFLP tissue had, on average, higher dsDNA content relative to SG (figure 3(B)).

Final dsDNA content in the automated ECM derived from either tissue was significantly reduced compared to native tissue, but not significantly different from ECM derived using the classical sheet method suggesting final DNA compliance in the quality of the decellularized materials. H&E histology analysis (figure 3(C)) shows a reduction of intact nuclei when comparing native vs decellularized tissues for either VFLP-ECM or SG-ECM. This result aligned with the DNA removal quantification within the final ECM shown in figure 3(B) (nested plot). Furthermore, muscle fibers and cytoplasm (Trichrome staining, in red) from VFLP showed improved preservation in the final ECM compared to the SG tissue. Collagen density within the materials (in blue) was well preserved in both tissues.

### 3.4. auVFLP-ECM vs auSG-ECM for the derivation of a VF targeted injectable ECM hydrogel

#### 3.4.1. Rheologic properties from solubilized ECM

**Figure 4.**
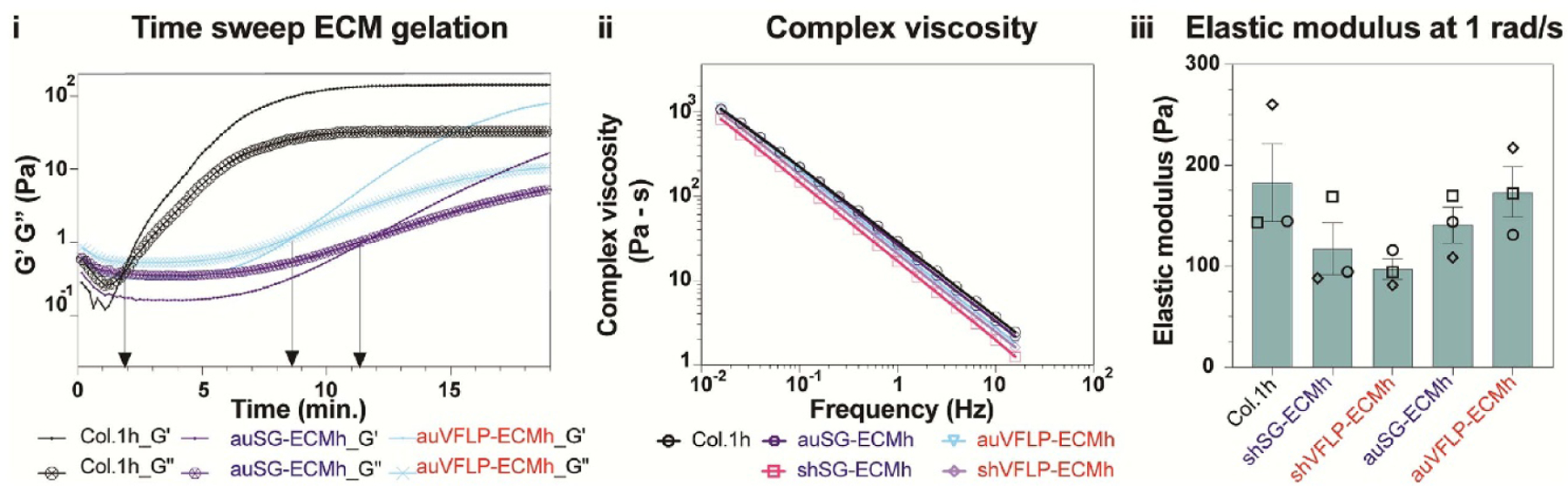
(I) Representative curves showing the gelation kinetics of various ECM samples (Col.1 h, auSG-ECMh, auVFLP-ECMh) plotted in terms of the elastic (G’) and viscous (G”) modulus. (ii) Complex viscosity vs frequency of ECM gels, (iii) Elastic modulus (G’) of ECM gels at 1 rad s^−1^.

Rheological properties of the derived ECM hydrogels were quantified during controlled temperature conditions. The evolution of elastic (G’) and viscous (G”) moduli as a function of time once 37 °C was reached is shown in figure 4(i). For all samples, both moduli increased with time; G’ increased more rapidly and eventually crossed over G”. This cross-over time was considered the approximate gel time [26]. Figure 6(i) also shows that Col.1 h had the shortest gel time, (2.06 ± 0.19 min). Longer moduli cross-over times were observed for auVFLP-ECMh and auSG-ECMh (8.79 ± 0.70 min and 11.12 ± 1.17 min, respectively).

Figure 4(ii) shows the complex viscosity (*) of all samples, plotted as a function of frequency. These data can be fitted to a power–law relation, as shown in equation (1) with n representing the power-law exponent

(1) $$\eta^{*}=kf^{-n}.$$

In the equation, η* is the complex viscosity given in Pa-s, *f* is frequency in Hz, and k and n are constants. The best set of data among the triad were fitted to equation (1) by simple linear regression and the data are summarized in table 2. Along with *k* and *n* values, *R*^2^ values are also provided to show the efficacy of the fit.

Elastic modulus of all ECM samples was obtained at a frequency of 1 rad s^−1^ (upon an equilibrated state) are shown in figure 4(iii). Col.1 h, which exhibited a gel modulus of 182.86 ± 38.72 Pa, was the ‘stiffer’ of the samples. The stiffness of auVFLP-ECMh (173.48 ± 24.88 Pa) and auSG-ECMh (140.85 ± 17.71 Pa) were slightly lower. shVFLP-ECMh (115.97 ± 10.12 Pa) and shSG-ECMh (94.48 ± 25.97 Pa) hydrogels were the ‘softest’.

#### 3.4.2. Discovery proteomics for automated derived ECM

**Figure 5.**
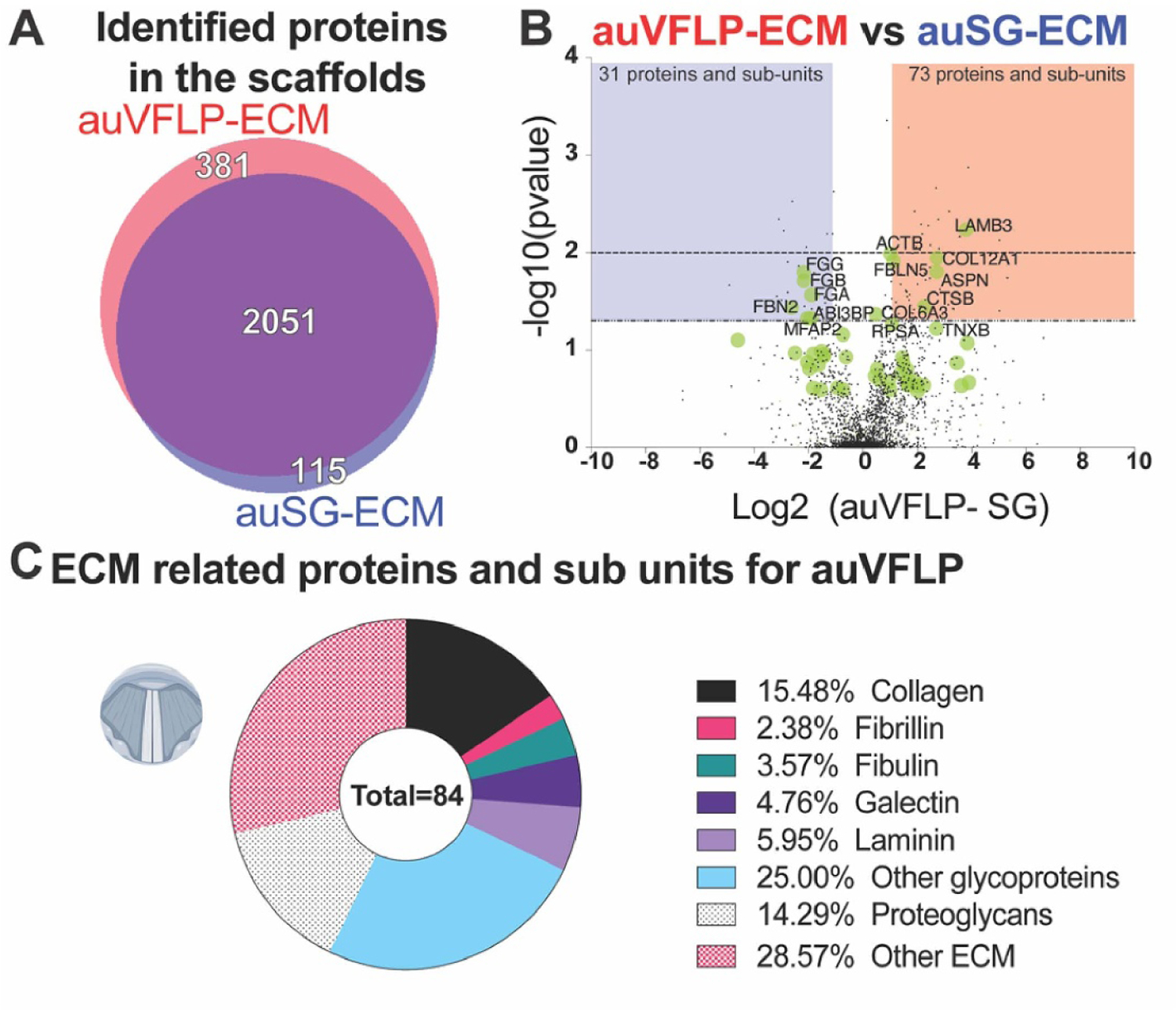
A. Venn diagram showing the number of unique proteins identified in auVFLP-ECM (red), auSG (blue). The number of common proteins identified for both material is quantified within the intersected area colored on purple. B. Volcano plot of the Log2 fold change (FC) of the ratio between auVFLP and auSG as a function of the p-values. The blue and red region were delimited by *p* value < 0.05 and a FC of at least *±*2 (*n* = 3). The points representing ECM associated proteins were colored on green and those protein with a *p* value < 0.05 were label with its corresponding gene ID. C. Pie chart for the auVLFP-ECM disclosing the proteins sub-unites identified and associated with ECM.

Protein composition of auVFLP-ECM and auSG-ECM were analyzed to characterize potential local tissue composition for VF applications. Proteomic discovery identified ∼2430 total proteins and sub-units within the auVFLP-ECM contrasted with 2160 proteins in auSG-ECM (figure 5(A)).

A large number of proteins (381 proteins) were uniquely identified for the auVFLP contrasted with 115 proteins in auSG. Figure 5(B) shows fold change analysis using Log2 of the abundance difference between auVFLP and auSG-ECM for the 2051 detected proteins. The significance threshold was delimited by an absolute fold change greater than 2 and a *p*-value lower than either 0.05 or 0.01. Thirty-one proteins were in higher abundance in auSG-ECM. In addition, there were 73 proteins contained in a larger quantity in auVFLP-ECM than in auSG-ECM. Data-points for proteins GO-identified within the protein class group of ECM proteins were indicated in green and labeled by name in the plot.

Figure 5(C) shows the auVFLP-ECM overall composition of identified ECM-related proteins; collagen was the third most representative group (figure S6 presents Log2 fold change bar plot using LFQ

### 3.5. auVFLP-ECMh effect in Macrophage (MΦs)

**Figure 6.**
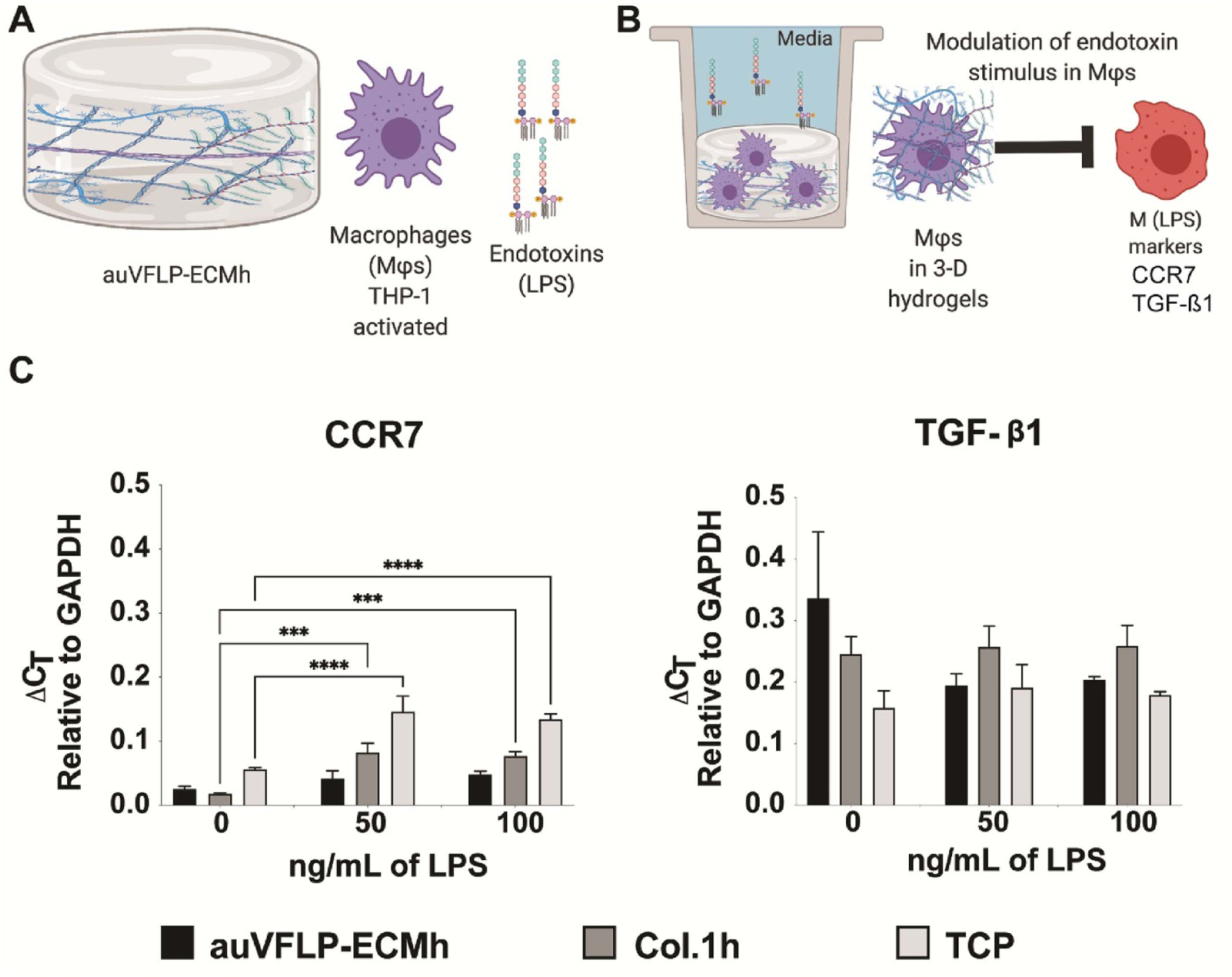
A. Overview schematics for the biomaterial testing comprised of auVFLP, THP-1 differentiated macrophages (MΦs), and endotoxins stimulation. B. Workflow depicting MΦs cultured in 3D with the ECM-hydrogels (auVFLP-ECM or Col.1h) upon LPS stimulation. C. RT-qPCR data for CCR7, associated with M1-like phenotype, and TGF-*β*1 gene associated with M2 like-phenotype. The error bars represent SEM, * = *p* value< 0.05, (*n* = 3).

Endotoxins (LPS) are commonly used *in vitro* to polarize MΦs towards a pro-inflammatory phenotype [21, 22, 27, 28]. LPS titration was used to assess expression of inflammatory-related markers in MΦs cultured on auVFLP-ECMh, Col1.h, and TCP (figures 6(A) and (B)).

THP1 MΦs were cultured with LPS for 48 h, and CCR7 (inflammatory marker) was measured via RT-qPCR. Also, TGF-*β*1 was included due to its role in fibrosis (all genes are shown in figure 6(C)). Increased expression of CCR7 was observed at all concentrations of LPS in MΦs cultured on Col.1h and TCP, but not in cells cultured on auVFLP-ECMh. The difference in expression of Col1.h and TCP was not statistically significant between 50 and 100 ng ml^−1^ of LPS. These data suggest that the expression of CCR7 can be modulated by the auVFL-ECM environment. TGF-*β*1 expression in MΦs on all the materials was not significantly different.

### 3.6. Optimizing the exposure time via monitoring the Abs. 260 nm to derived auVFLP-ECM

**Figure 7.**
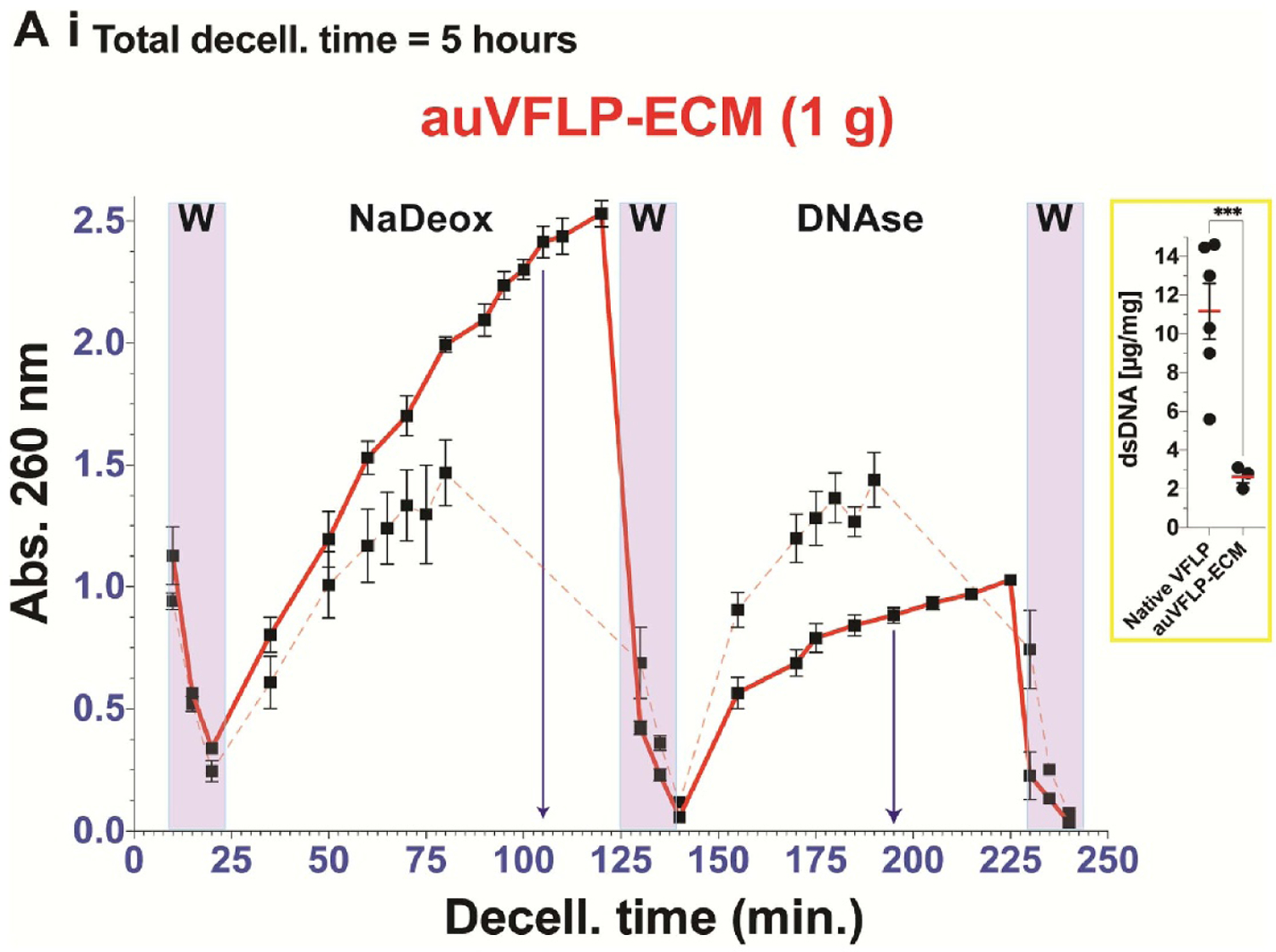
A (i) Monitoring profile obtained for the automated decellularization method for 1 gr of auVFLP tissue, following the protocol listed in table S1 B(iii) decellularization stages (W = wash and reag. = reagents a, b, or c). Dash profile represents the superposition of the monitoring curve obtained for the tissue under the 4 h protocol as depicted in figure 3(B)ii, table S1 B(ii), for auVFLP-ECM. The nested sub-plot shows dsDNA content quantification for VFLP tissue and decellularized ECM (native and ECM). The error bars represent the SEM (*n* = 3), *p < 0.05.

VFLP (1 g) was exposed for 100 min per reagent (i.e. NaDeox and DNAse) to evaluate the highest peak and plateau in abs.260 nm, particularly for the NaDeox phase. The monitoring profile is shown in figure 7(i).

Overall, an intensity peak was observed ∼1.6× higher for the NaDeox stage after 100 min of treatment compared to 60 min (dashed profile superposed from figure 3(Bii) for auVFLP). This outcome suggests extended exposure time to NaDeox led to increased removal of nucleic acids or other analytes capable of absorbing the spectroscopic signal. During the last 10 min of detergent exposure, a lower slope in the signal trend was observed, which may indicate a plateau. For DNAse, the curve depicted a linear, incremental trend for the first 20–30 min of treatment and a considerable reduction in the signal after 60 min.

## 4. Discussion

Decellularized ECM scaffolds are of interest to the research and clinical communities, given their unique regenerative and cell-modulatory properties [10]. The decellularization process mainly focuses on the removal of unwanted immunogenic molecules within the tissue while preserving other bioactive components. A variety of decellularization methods have been developed, in part, due to the need for tissue-specific adjustments and lab-specific requirements. These protocol adjustments, however, likely limit standardization and optimization of effective protocols across labs and during commercial manufacturing [29]. Although reports correlate the efficiency of decellularization with the reagents used and their exposure times, this association remains poorly understood [30, 31]. A robust tool/technique to track the effectiveness of decellularization could facilitate the study of relevant process parameters that impact the effectiveness and efficiency of the process.

Geerts *et al* described a cell removal monitoring approach via computer tomography. However, the instrumentation required is not readily available in labs, could be difficult to translate to larger-scale manufacturing, and adds high costs to the entire process [32]. Final DNA content within the decellularized ECM is commonly used as an end-measurement to evaluate the effectiveness of the decellularization process. Although specific dsDNA kits are currently used to measure the final amount of DNA, the monitoring of DNA removal during the process using spectroscopic technics (widely available in laboratories) could become an important measuring tool [8, 33]. Developing a technique based on absorbance spectroscopy to monitor decellularization via tracing nucleic acid removal from the tissue is likely to facilitate the optimization of protocols and a platform to control efficiency.

The present study decellularized two model tissues from the upper airway to determine the usefulness of absorbance measurements for monitoring and optimizing decellularization. Although absorbance at 260 nm is an accepted measurement for nucleic acids detection and quantification, other analytes may contribute to the signal. DNA within the SG-tissue (native) was tagged using a permeable nuclear fluorophore (Hoechst 33258) prior to decellularization (figure S3(A)) to determine the specific contribution of DNA to the tracing signal. Absorbance and reagent fluorescence values, measured from the bioreactor effluent, were plotted as a function of exposure time (figure s3(Bi)). The trend between absorbance and Hoechst-fluorescence curves were analogous using NaDeox supporting our observation that DNA contributes to the signal. Values at every point for fluorescent profile during DNAse treatment (region b) were substantially lower than absorbance during the same stage. This difference in spectroscopy readings (Fluorescence vs. Abs. 260 nm during the DNAse treatment) aligns with findings from Vekshin *et al*, which showed lower fluorescence by DNA-Hoechst complex when hydrolyzed with DNAse [34]. DNA-hydrolysis via the DNAse nuclease reaction affected the fluorescent signal but did not hinder absorbance tracing, likely due to the increased absorption capacity of nucleotides compared to DNA or RNA strands [35]. This finding enabled us to build release profiles or a combination between release profile and nucleotide concentration in solution, as shown in figures 2, 3, and 7. To the best of our knowledge, these profiles are the first characterization of decellularization dynamics and warrants further investigation.

One of the advantages of reading absorbance values over time is that they can be applied to traditional decellularization methods (manual methods). When using the nucleic acid tracking technique for the classical sheet (sh) batch method to decellularize both shVFLP and shSG (figure 2(B)), the profile for nucleic acids in solution resembled a cumulative release plot where the absorbance (concentration) increased as a function of reagent exposure time. The shVFLP profile had higher absorbances at every time point compared to shSG during the detergent (NaDeox) stage, suggesting either higher initial DNA content or higher nucleic acid release from the VFLP to the liquid phase when compared to SG (or a combination of both factors). This finding is an example of how this technique can be used to compare the decellularization of two tissues using the same protocol. This process may help predict the final decellularization outcomes as well as optimize the final product by improving our understanding of the dynamics during the decellularization process at each step.

Absorbance readings can also be used inline during automated processes to detect differences in the tissue or sample preparation by comparing release profiles. Profiles obtained for automated decellularization showed higher intensity in the signal for VFLP decellularization compared to SG, supporting the notion that this technique can detect differences in the initial nucleic acid content between tissues. When quantifying initial dsDNA content within the native tissue, VFLP had more dsDNA than SG on average. The correlation between the quantity of tissue loaded into the bioreactor and signal intensity is shown for VFLP in figure S2. These data suggest the method can be used to monitor higher DNA concentrations under the parameters tested. When comparing across decellularization methods (figures 2(Bi), 3(Bii), and 7 for the VFLP), the au-method showed higher absorbance values at every time point than the classical sh-method. It is important to highlight that the same reagents were used, but shorter exposure times were implemented for the automated method. Higher release of dsDNA in the au method may be related to the smaller material size (larger available area) in combination with the agitation in the bioreactor. End-point testing such as dsDNA content (nested plots) and histology corroborate the significantly reduced dsDNA content and the absence of intact nuclei within the decellularized ECM. This finding demonstrates monitoring absorbance can be used to compare manual and automated methods, and that this information can be used to further improve each process.

One of the goals of decellularizing tissues is to have a clinically feasible material. ECM scaffolds can be transformed into an injectable hydrogel to be delivered via minimally invasive methods to the site of injury. We focused on potential applications for airway indications where hydrogels can be injected into injured VFs to reduce scarring and promote tissue regeneration. Characterization of rheological and bulking properties of ECM hydrogels is critical to determine clinical utility via injection. The time-dependent behavior of the moduli of all samples (figure 4(i)) was a stage where G” initially dominated (i.e. had higher values) G’. During this stage, samples were in a solution state (confirmed by steady shear viscosity, data not shown). With increased time, the samples underwent gelation with the cross-over point reflecting an approximate gel time. A true gel point, based on the Winter-Chambon criteria, is not appropriate for the current study but will be considered in future work [36]. Eventually, moduli values tended to plateau with G’ dominating G” as they reached a gel state. Gelation occurs as a result of the self-assembly of proteins and polysaccharides inside ECM matrices [20]. We observed a slightly delayed gelation time for auVFLP-ECMh and auSG-ECMh compared to Col.1h. This delay could be attributed to interactions between ECM contents such as laminin, galectin, and proteoglycans. The volcano plot in figure 5(B) shows that auVFLP-ECMh had a higher content of some collagen subunits compared to auSG-ECMh, but not all were significant. Therefore, the slightly faster gelation of auVFLP-ECMh compared to auSG-ECMh may be related to protein content, which could affect the self-assembly of collagen fibers during gelation [37]. Although collagen is a well-established hydrogel, fast gelation may not always be desirable as it may create clinical difficulties during injection. Delayed gelation of auVFLP-ECMh and auSG-ECMh may provide improved delivery and spread at the location of the application [38].

Injectability of hydrogels for airway repair is often characterized via complex viscosity plotted as a function of frequency and fitted to a linear equation. Figure 4(ii) shows the complex viscosity of all samples to show a power-law behavior with respect to frequency. We observed a slope close to −1 and an absence of a Newtonian plateau, consistent with gel-like materials [39]. Similar shear-thinning behavior has been observed in other ECM hydrogels [40]. The *k* values obtained by fitting the data show that auSG-ECMh and Col.1h had similar viscosities. auVFLP-ECMh had a slightly lower viscosity, which may be due to limited interaction sites of collagen molecules by other components inside the ECM [40]. Gel elastic modulus values of auVFLP-ECMh and auSG-ECMh hydrogels were similar to collagen (figure 4(iii)). The auSG-ECMh hydrogel appeared slightly softer than auVFLP-ECMh based on lower elastic modulus. Higher content of fibrillin in auSG-ECMh may contribute to this lower modulus given the flexible nature of fibrillin proteins, but further targeted protein composition studies are needed [41, 42]. The auVFLP-ECMh showed a higher gel modulus when compared to Col.1h, suggesting a higher proportion of components, including other collagens. Of note, complex viscosity results (figure 4(ii)) reflect both elastic and viscous contributions to the rheological response, whereas the modulus data in figure 4(iii) are related to elasticity from the effective network junction. A sample with the smallest k value does not necessarily possess the lowest modulus. In general, we anticipated rheological properties would also be affected by decellularization or ECM-solubilization such as reagent sequence, exposure time, changes in pH, temperature, and salt concentration during ECM digestion [43, 44]. It is encouraging that, despite longer gelation times, the properties of VFLP and SG hydrogels obtained through the automated process were similar to Col.1h when compared to samples decellularized with the classical sh-method. Comparable rheological properties of the ECM gels produced via the automated processes indicate potential utility as injectable materials.

The unique protein composition and the rheological properties made auVFLP a strong candidate for *in vitro* characterization. Given the known ability of ECM scaffolds to modulate macrophage polarization [45], we tested the effects of auVFLP hydrogel upon *in vitro* polarization when challenged with LPS. When differentiated THP-1 MΦs were cultured on Col.1h (the hydrogel control) and TCP, increased CCR7 (classical marker associated with inflammatory phenotypes) expression was observed. auVFLP-ECMh, without LPS, did not stimulate the expression of CCR7. Also, no statistically significant increase in CCR7 expression was observed when macrophages were grown in auVFLP-ECMh and exposed to higher concentrations of LPS. This overall response suggests a potential modulatory effect of auVFLP-ECM upon THP-1 MΦs. Interestingly, we did not measure differences in TGF-*β*1 expression after LPS stimulation, a cytokine linked to fibrosis in VF fibroblasts. The effect of the ECM biomaterial on TGF-*β*1 requires further investigation.

Given the potential modulatory properties of auVFLP-ECM on macrophages, we used the monitoring tool described in this study to optimize VFLP decellularization using the auVFLP method. We approached the decellularization optimization by seeking to increase nucleic acid release during the detergent stage via longer exposure times (figure 7(Ai)) when compared to the initial protocol used (monitoring curve shown in figure 3(Bii). We used 1 g of VFLP during an extended exposure time of 100 min of each reagent (i.e. NaDeox and DNAse) for a total of 5 h. The protocol is listed in table S1(Biii), and the monitoring profile is shown in figure 7(Ai) where the dashed monitoring profile within the plot represents the superimposed profile from figure 3(Bii) to facilitate comparisons. As expected, the highest values were obtained in response to NaDeox, which likely reduced the quantity of DNA available for subsequent DNAse treatment, resulting in a lower intensity profile. This finding suggests that an adequate exposure time for NaDeox may be between 80 and 90 min with a DNAse exposure of 30–50 min for 1 g of starting ECM. These results serve as an example of how this tool can be used to obtain critical process information of this complex process to optimize decellularization protocols.

Interestingly, as shown in figure 7(Ai), increased exposure modified the intensity trends of the curves between stages (i.e. the absorbance for the NaDeox was significantly higher than DNAse). This result may support decellularization mechanism(s) where the detergent penetrates the tissue disrupting cells and nuclear membranes, but exposure time and/or agitation contribute to detachment of larger DNA strands from the ECM. Also, the release profiles for DNAse steps suggest DNA strands remained available for nuclease hydrolysis facilitating the release from the tissue into the liquid phases. Figures 2 and 3 show analogous intensity for the release profiles between NaDeox and DNAse. However, figure 7 shows a shift in intensity between the detergent and nuclease steps. This raises an important point regarding the effects of shifting DNA release from the nuclease stage to the detergent stage and how this shift impacts the materials’ regenerative properties. This shift is important because the balance between reagent sequence, time of exposure, and ECM biomaterial properties remains poorly understood.

It is important to note that the technique is limited to nucleic acid tracing (e.g. DNA), and other macromolecules may not be detected or affect the signal. Therefore, other inline spectroscopy tools could be tested in future studies to evaluate the potential use of monitoring other key components during decellularization. Another limiting factor for this technique is the requirement of a low UV background from the reagents or solution. For example, the combination of NaDeox and TE (figure S4) interfered with the linearity of lower DNA concentrations suggesting that the solution should be evaluated prior to tracing nucleic acid. Nevertheless, a tool to monitor and promote faster DNA removal is promising and can be incorporated into a fully automated system. The monitoring method has the potential to be applied to a large number of decellularization protocols used to obtain ECM scaffolds from multiple tissues. Data from the present study provide a foundation to adjust decellularization parameters to promote an increased release of DNA and guide and optimize decellularization protocols.

## 5. Conclusion

Absorbance spectroscopy at 260 nm can be used to monitor decellularization processes by tracing nucleic acid release from tissues to the liquid phase (i.e. bioreactors effluent) as a function of reagent exposure time. The derived ECMs (VFLP or SG), when solubilized and gelled, produced a biomaterial whose rheological properties resemble other ECM hydrogels used for VF applications. Also, the auVFLP-ECM gel modulated the cell behavior of differentiated THP-1 MΦs upon LPS stimulation. The developed spectroscopy approach enables optimizing decellularization protocols in real-time, advancing the field towards a controlled process for either semi-batch (au) or batch (sh) derivation of VF-specific ECM scaffolds as well as other soft tissues.

## Supplementary Material

Supplemental Material

## Figures and Tables

**Table 1.** RT-qPCR Primers.

Gene	Forward sequence	Reverse sequence
GAPDH	AAGGTGAAGGTCGGAGTCAAC	GGGGTCATTGATGGCAACAATA
CCR7	TGAGGTCACGGACGATTACAT	GTAGGCCCACGAAACAAATGAT
TGF-*β*1	GGCCAGATCCTGTCCAAGC	GTGGGTTTCCACCATTAGCAC

**Table 2.** Comparison of the complex viscosity of ECM hydrogels.

ECMs	Gelation time (min)	*k*	*n*	*R* ^2^
Col.1h	2.06 *±* 0.19	31.4	0.854	0.99
auVFLP-ECMh	8.79 *±* 0.70	24.99	0.917	0.99
auSG-ECMh	11.12 *±* 1.17	32.02	0.849	0.99
shVFLP-ECMh	4.14 *±* 0.24	22.12	0.915	0.99
shSG-ECMh	6.12 *±* 0.85	17.35	0.93	0.99

## Data Availability

All data that support the findings of this study are included within the article (and any supplementary files).

## References

[1] Kabirian F and Mozafari M 2020 Decellularized ECM-derived bioinks: prospects for the future Methods 171 108–18 31051254 10.1016/j.ymeth.2019.04.019

[2] Saldin LT, Cramer MC, Velankar SS, White LJ and Badylak SF 2017 Extracellular matrix hydrogels from decellularized tissues: structure and function Acta Biomater 49 1–15 27915024 10.1016/j.actbio.2016.11.068 PMC5253110

[3] Traverse JH 2019 First-in-man study of a cardiac extracellular matrix hydrogel in early and late myocardial infarction patients JACC 4 650–69 10.1016/j.jacbts.2019.07.012 PMC6834965 31709316

[4] Hoshiba T and Tanaka M 2016 Decellularized matrices as *in vitro* models of extracellular matrix in tumor tissues at different malignant levels: mechanism of 5-fluorouracil resistance in colorectal tumor cells Biochim. Biophys. Acta 1863 2749–57 27558478 10.1016/j.bbamcr.2016.08.009

[5] O’Neill JD, Freytes DO, Anandappa AJ, Oliver JA and Vunjak-Novakovic GV 2013 The regulation of growth and metabolism of kidney stem cells with regional specificity using extracellular matrix derived from kidney Biomaterials 34 9830–41 24074840 10.1016/j.biomaterials.2013.09.022 PMC3835733

[6] Smith LR, Cho S and Discher DE 2018 Stem cell differentiation is regulated by extracellular matrix mechanics Physiology 33 16–25 29212889 10.1152/physiol.00026.2017 PMC5866410

[7] McCrary MW, Bousalis D, Mobini S, Song YH and Schmidt CE 2020 Decellularized tissues as platforms for *in vitro* modeling of healthy and diseased tissues Acta Biomater 111 1–19 32464269 10.1016/j.actbio.2020.05.031

[8] Aamodt JM and Grainger DW 2016 Extracellular matrix-based biomaterial scaffolds and the host response Biomaterials 86 68–82 26890039 10.1016/j.biomaterials.2016.02.003 PMC4785021

[9] Hussey GS, Dziki JL and Badylak SF 2018 Extracellular matrix-based materials for regenerative medicine Nat. Rev. Mater 3 159–73

[10] Cramer MC and Badylak SF 2019 Extracellular matrix-based biomaterials and their influence upon cell behavior Ann. Biomed. Eng 48 2132–53 31741227 10.1007/s10439-019-02408-9 PMC7231673

[11] Hernandez MJ 2019 Manufacturing considerations for producing and assessing decellularized extracellular matrix hydrogels Methods 171 20–27 31546012 10.1016/j.ymeth.2019.09.015

[12] Pereira RHA, Prado AR, Caro L, Zanardo TEC, Alencar AP and Nogueira BV 2019 A non-linear mathematical model using optical sensor to predict heart decellularization efficacy Sci. Rep 9 12211 31434981 10.1038/s41598-019-48659-3 PMC6704168

[13] Badileanu A 2020 Fast automated approach for the derivation of acellular extracellular matrix scaffolds from porcine soft tissues Acs Biomater. Sci. Eng 6 4200–13 33463339 10.1021/acsbiomaterials.0c00265 PMC8133378

[14] Schmid F-X 2001 Biological Macromolecules: UV-visible Spectrophotometry (Chichester, UK: John Wiley & Sons, Ltd) pp 1–4

[15] Wrona EA, Peng R, Born H, Amin MR, Branski RC and Freytes DO 2016 Derivation and characterization of porcine vocal fold extracellular matrix scaffold Laryngoscope 126 928–35 26371887 10.1002/lary.25640

[16] Wisniewski JR, Zougman A, Nagaraj N and Mann M 2009 Universal sample preparation method for proteome analysis Nat. Methods 6 359–U60 19377485 10.1038/nmeth.1322

[17] Hulsen T, de Vlieg J and Alkema W 2008 BioVenn—a web application for the comparison and visualization of biological lists using area-proportional Venn diagrams BMC Genomics 9 488 18925949 10.1186/1471-2164-9-488 PMC2584113

[18] Mi H 2019 Protocol Update for large-scale genome and gene function analysis with the PANTHER classification system (v.14.0) Nat. Protoc 14 703–21 30804569 10.1038/s41596-019-0128-8 PMC6519457

[19] Naba A, Clauser KR, Ding H, Whittaker CA, Carr SA and Hynes RO 2016 The extracellular matrix: tools and insights for the “omics” era Matrix Biol 49 10–24 26163349 10.1016/j.matbio.2015.06.003 PMC5013529

[20] Freytes DO, Martin J, Velankar SS, Lee AS and Badylak SF 2008 Preparation and rheological characterization of a gel form of the porcine urinary bladder matrix Biomaterials 29 1630–7 18201760 10.1016/j.biomaterials.2007.12.014

[21] Spiller KL 2016 Differential gene expression in human, murine, and cell line-derived macrophages upon polarization Exp. Cell Res 347 1–13 26500109 10.1016/j.yexcr.2015.10.017

[22] Wrona EA, Sun B, Romero-Torres S and Freytes DO 2019 Effects of polarized macrophages on the *in vitro* gene expression after Co-Culture of human pluripotent stem cell-derived cardiomyocytes J. Immunol. Regener. Med 4 100018

[23] Bruyneel AAN and Carr CA 2017 Ambiguity in the presentation of decellularized tissue composition: the need for standardized approaches Artif. Organs 41 778–84 27925237 10.1111/aor.12838 PMC5600108

[24] Fernandez-Perez J and Ahearne M 2019 Author correction: the impact of decellularization methods on extracellular matrix derived hydrogels Sci. Rep 9 19818 31852982 10.1038/s41598-019-56283-4 PMC6920349

[25] Gilbert TW, Freund JM and Badylak SF 2009 Quantification of DNA in biologic scaffold materials J. Surg. Res 152 135–9 18619621 10.1016/j.jss.2008.02.013 PMC2783373

[26] Corder RD, Dudick SC, Bara JE and Khan SA 2020 Photorheology and gelation during polymerization of coordinated ionic liquids ACS Appl. Polym. Mater 2 2397–405

[27] Spiller KL 2014 The role of macrophage phenotype in vascularization of tissue engineering scaffolds Biomaterials 35 4477–88 24589361 10.1016/j.biomaterials.2014.02.012 PMC4000280

[28] Mora-Navarro C, Ozpinar EW, Sze D, Martin DP and Freytes DO 2021 Transcriptome-targeted analysis of human peripheral blood-derived macrophages when cultured on biomaterial meshes Biomed. Mater 16 025006 33445160 10.1088/1748-605X/abdbdb PMC11626613

[29] Emami A, Talaei-Khozani T, Vojdani Z and Zarei Fard N 2021 Comparative assessment of the efficiency of various decellularization agents for bone tissue engineering J. Biomed. Mater. Res. B 109 19–32 10.1002/jbm.b.34677 32627321

[30] Lee PF 2017 Inverted orientation improves decellularization of whole porcine hearts Acta Biomater 49 181–91 27884776 10.1016/j.actbio.2016.11.047

[31] McCrary MW, Vaughn NE, Hlavac N, Song YH, Wachs RA and Schmidt CE 2020 Novel sodium deoxycholate-based chemical decellularization method for peripheral nerve Tissue Eng. C 26 23–36 10.1089/ten.TEC.2019.0135 31724493

[32] Geerts S, Ozer S, Jaramillo M, Yarmush ML and Uygun BE 2016 Nondestructive methods for monitoring cell removal during rat liver decellularization Tissue Eng. C 22 671–8 10.1089/ten.tec.2015.0571 PMC4943465 27169332

[33] Wong ML and Griffiths LG 2014 Immunogenicity in xenogeneic scaffold generation: antigen removal vs. decellularization Acta Biomater 10 1806–16 24486910 10.1016/j.actbio.2014.01.028 PMC3976714

[34] Vekshin NL, Doynikova AN and Lvov AM 2019 Determination of micro-quantities of DNA using DNAse and fluorescence of hoechst 33258 and light-scattering J. Fluoresc 29 479–84 30811018 10.1007/s10895-019-02358-4

[35] Laukova L, Konecna B, Janovicova L, Vlkova B and Celec P 2020 Deoxyribonucleases and their applications in biomedicine Biomolecules 10 1036 10.3390/biom10071036 PMC7407206 32664541

[36] Chambon F and Winter HH 1987 Linear viscoelasticity at the gel point of a crosslinking PDMS with imbalanced stoichiometry J. Rheol 31 683–97

[37] Toprakhisar B, Nadernezhad A, Bakirci E, Khani N, Skvortsov GA and Koc B 2018 Development of bioink from decellularized tendon extracellular matrix for 3D bioprinting Macromol. Biosci 18 e1800024 30019414 10.1002/mabi.201800024

[38] Choi YH, Kim SH, Kim IG, Lee JH and Kwon SK 2019 Injectable basic fibroblast growth factor-loaded alginate/hyaluronic acid hydrogel for rejuvenation of geriatric larynx Acta Biomater 89 104–14 30849562 10.1016/j.actbio.2019.03.005

[39] Farias BV, Hsiao LC and Khan SA 2020 Rheological and tribological behavior of gels and emulsions containing polymer and phospholipid ACS Appl. Polym. Mater 2 1623–33

[40] Chan RW and Titze IR 1998 Viscosities of implantable biomaterials in vocal fold augmentation surgery Laryngoscope 108 725–31 9591554 10.1097/00005537-199805000-00019

[41] Muiznieks LD and Keeley FW 2013 Molecular assembly and mechanical properties of the extracellular matrix: a fibrous protein perspective Biochim. Biophys. Acta 1832 866–75 23220448 10.1016/j.bbadis.2012.11.022

[42] Sakai LY, Keene DR and Engvall E 1986 Fibrillin, a new 350-kD glycoprotein, is a component of extracellular microfibrils J. Cell Biol 103 2499–509 3536967 10.1083/jcb.103.6.2499 PMC2114568

[43] Johnson TD, Lin SY and Christman KL 2011 Tailoring material properties of a nanofibrous extracellular matrix derived hydrogel Nanotechnology 22 494015 22101810 10.1088/0957-4484/22/49/494015 PMC3280097

[44] Xu CC, Chan RW and Tirunagari N 2007 A biodegradable, acellular xenogeneic scaffold for regeneration of the vocal fold lamina propria Tissue Eng 13 551–66 17518602 10.1089/ten.2006.0169

[45] Brown BN, Valentin JE, Stewart-Akers AM, McCabe GP and Badylak SF 2009 Macrophage phenotype and remodeling outcomes in response to biologic scaffolds with and without a cellular component Biomaterials 30 1482–91 19121538 10.1016/j.biomaterials.2008.11.040 PMC2805023

