# Supplementary material for: Monitoring decellularization via absorbance spectroscopy during the derivation of extracellular matrix scaffolds
Source: Biomed Mater. Author manuscript; Available in PMC 2022 Aug 26. (PMC9416610; doi:10.1088/1748-605X/ac361f)
Supplement: Supplemental Material [file NIHMS1828469-supplement-Supplemental_Material.pdf]

Monitoring decellularization via absorbance spectroscopy during the derivation of extracellular matrix scaffolds

Camilo Mora-Navarro[1,2,7], Mario Garcia[1], Prottasha Sarker[3], Emily W. Ozpinar[1,2], Jeffrey R. Enders[4,5], Saad Khan[3], Ryan C. Branski[6] and Donald O. Freytes[1,2]

[1]Joint Department of Biomedical Engineering, North Carolina State and University of North Carolina-Chapel Hill, Raleigh, NC, United States of America

[2]Comparative Medicine Institute, North Carolina State University, Raleigh, NC, United States of America

[3] Department of Chemical and Biomolecular Engineering, North Carolina State University, Raleigh, NC 27695, United States of America

[4]Molecular Education, Technology and Research Innovation Center, North Carolina State University, Raleigh, NC, United States of America

[5]Department of Biological Sciences, North Carolina State University, Raleigh, NC, United States of America

[6]Departments of Rehabilitation Medicine and Otolaryngology-Head and Neck Surgery, NYU Grossman School of Medicine, New York, NY, United States of America

[7]Department of Chemical Engineering, University of Puerto Rico, Mayaguez, PR, United States of America

Email: dofreyte@ncsu.edu

Keywords: Extracellular Matrix, Manufacturing, Decellularization, Monitoring, Vocal fold

Figure 1. Project overview. i) Workflow for at process determination of the absorbance at 260 nm from the bioreactor's effluent. ii) Derived extracellular matrix (ECM) biomaterial characterization centered on an injectable scaffold for vocal fold application.

Figure 2. A i) Open view of the porcine laryngeal area showing VFLP and SG tissue before dissection. ii) VFLPs dissected and in the process of being decellularized using the classical sheet (sh) method. iii) Approach to plot the absorbance intensity at 260 nm from the samples taken as a function of the decellularization time per reagent stage. iv) Macroscopic view of the decellularized tissues after classical method. **B** i) Monitoring profiles obtained for the classical decellularization method for either VFLP or SG tissue per decellularization stage. The break in the x-axis represents an overnight storage step (not added to the total decellularization time). The nested sub-plots display the double strand DNA quantification for the native tissue and decellularized-ECM. The error bars represent the SEM (n=3), * p-values < 0.05.

Figure 3. A i) Open view of laryngeal area showing VFLP and SG tissue delimited before dissection. ii) VFLPs and SG native tissue (raw) minced in pieces < 3 mm before being load to the semi-batch bioreactor. iii) Automated decellularization station showing the semi-batch bioreactor and the dossing system. iv) Decellularization workflow showing the sequential decellularization stages (W= wash and reag. = reagents a, b, or c). Also, the figure shows the sample inline port used for aliquot collection. **B** i) Monitoring profiles obtained for the automated decellularization method for 0.5 gr of either VFLP or SG tissue, following the protocol listed in Table S1 B(i). ii) Monitoring profile obtained for the automated decellularization method for 1 gr of either VFLP or SG tissue, following the protocol listed in Table S1 B(ii). The nested sub-plots display the double strand DNA quantification for the native tissue and decellularized-ECM. **C** Histology staining of native tissue and automated decellularized ECM**.** The error bars represent the SEM (n=3), * p-values < 0.05.

Figure 4. i) Representative curves showing the gelation kinetics of various ECM samples (Col.1h, auSG-ECMh, auVFLP-ECMh) plotted in terms of the elastic (G') and viscous (G'') modulus. **ii)** Complex viscosity vs frequency of ECM gels, **iii)** Elastic modulus (G') of ECM gels at 1 rad/s.

Figure 5. A Venn diagram showing the number of unique proteins identified in auVFLP-ECM (red), auSG (blue). The number of common proteins identified for both material is quantified within the intersected area colored on purple. **B** Volcano plot of the Log2 fold change (FC) of the ratio between auVFLP and auSG as a function of the p-values. The blue and red region were delimited by pvalue < 0.05 and a FC of at least +/-2 (n=3). The points representing ECM associated proteins were colored on green and those protein with a pvalue < 0.05 were label with its corresponding gene ID**. C** Pie chart for the auVLFP-ECM disclosing the proteins sub-unites identified and associated with ECM.

Figure 6. A. Overview schematics for the biomaterial testing comprised of auVFLP, THP-1 differentiated macrophages (Mϕs), and endotoxins stimulation. **B.** Workflow depicting Mϕs cultured in 3-D with the ECM-hydrogels (auVFLP-ECM or Col.1h) upon LPS stimulation. C. RT-qPCR data for CCR7, associated with M1-like phenotype, and TGF-β1 gene associated with M2 like-phenotype. The error bars represent SEM, * = pvalues< 0.05, (n=3).

Figure 7. A i) Monitoring profile obtained for the automated decellularization method for 1 gr of auVFLP tissue, following the protocol listed in Table S1 B(iii) decellularization stages (W= wash and reag. = reagents a, b, or c). Dash profile represents the superposition of the monitoring curve obtained for the tissue under the 4 hours protocol as depicted in Figure 3Bii, Table S1 B(ii), for auVFLP-ECM. The **nested sub-plot shows** dsDNA content quantification for both VFLP and SG tissues (native and ECM). the error bars represent the SEM (n=3), * p<0.05.

Supporting Table S1. Decellularization steps:

**A.** sh(VFLP or SG)-ECM Protocol time (6 hours)			**B.** au(VFLP or SG) Protocol time (**i = 3 hours, ii = 4 hours & iii = 5 hours**)				
ID	Reagent	exposure time (min)	ID	Reagent	(i) exposure time (min)	(ii) exposure time (min)	(iii) exposure time (min)
		15		DI_Water	5	5	5
W	1XDPBS	15	W	1XDPBS	5	5	5
		15		DI_Water	5	5	5
Deterg.	NaDeox	120	Deterg.	NaDeox	30	60	100
				DI_Water	5	5	5
W	1XDPBS	15	W	2XDPBS	5	5	5
				DI_Water	5	5	5
Nuclea.	DNAse	120	Nuclea.	DNAse	30	60	100
				DI_Water	5	5	5
W	1XDPBS	15	W	2XDPBS	5	5	5
				DI_Water	5	5	5
Ster.	Perac.Ac	30	Ster.	Perac.Ac	30	30	30
		15		DI_Water	5	5	5
W	1XDPBS	15	W	2XDPBS	15	15	15
		15		DI_Water	5	5	5
	Total (min)	390		Total (min)	160	220	300
	(hours)	**6.5**		(hours)	**~3**	**~4**	**5**

VFLP = Vocal fold lamina propria
SG = Supraglottic
Sh = classical method sheet.
Au = automated method
Min = minutes
W = wash
DPBS = Dulbecco's phosphate buffered saline
Deterg. = Detergent
NaDeox = Sodium Deoxycholate
Nuclea = Nuclease
DNAse = DNAse A type I
Ster. = Sterilization

Perac. Ac. = Peracetic Acid
DI_water = Deionized Water

Figure S1. Overnight storage

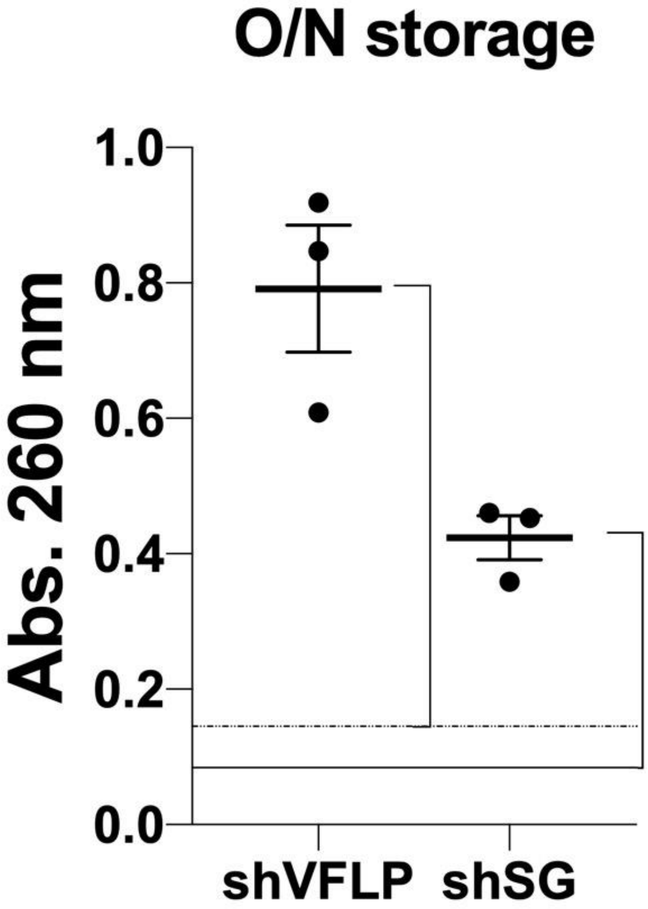

The O/N condition represents the tissue stored overnight at 4 ^{o}C in DPBS 1%Anti/Anti. This O/N time is not added to the total protocol time, but it is part of the protocol to complete the last two decellularization steps sequentially (non-stop) during the same day. The error bars represent the SEM (n=3). (------- absorbance measured before O/N storage for VFLP, —— absorbance measured before O/N storage for SG) .

Figure S2. Signal intensity in the function of the Initial mass fed to the bioreactor

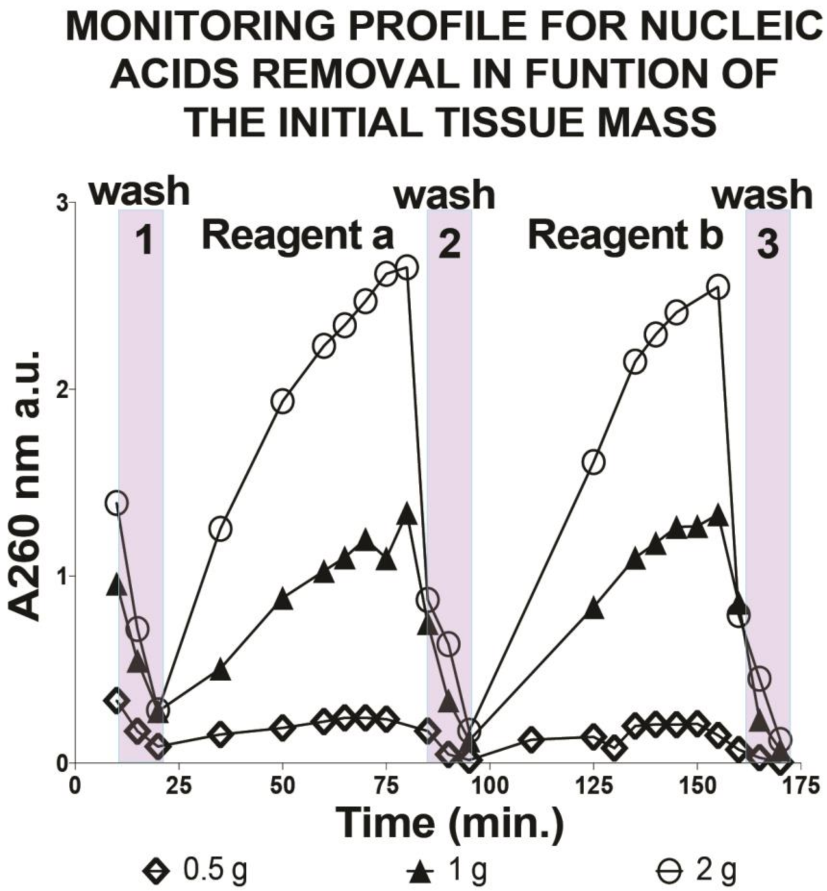

A. Monitoring profiles obtained for three initial quantities of tissue loaded and decellularized via automated method, following the protocol listed in Table S1 B(ii) decellularization stages (Reagent a = NaDEox 4%; Reagent b = DNAseA).

Figure S3. Hoechst dsDNA pre-stained and correlation with Abs. 260nm signal

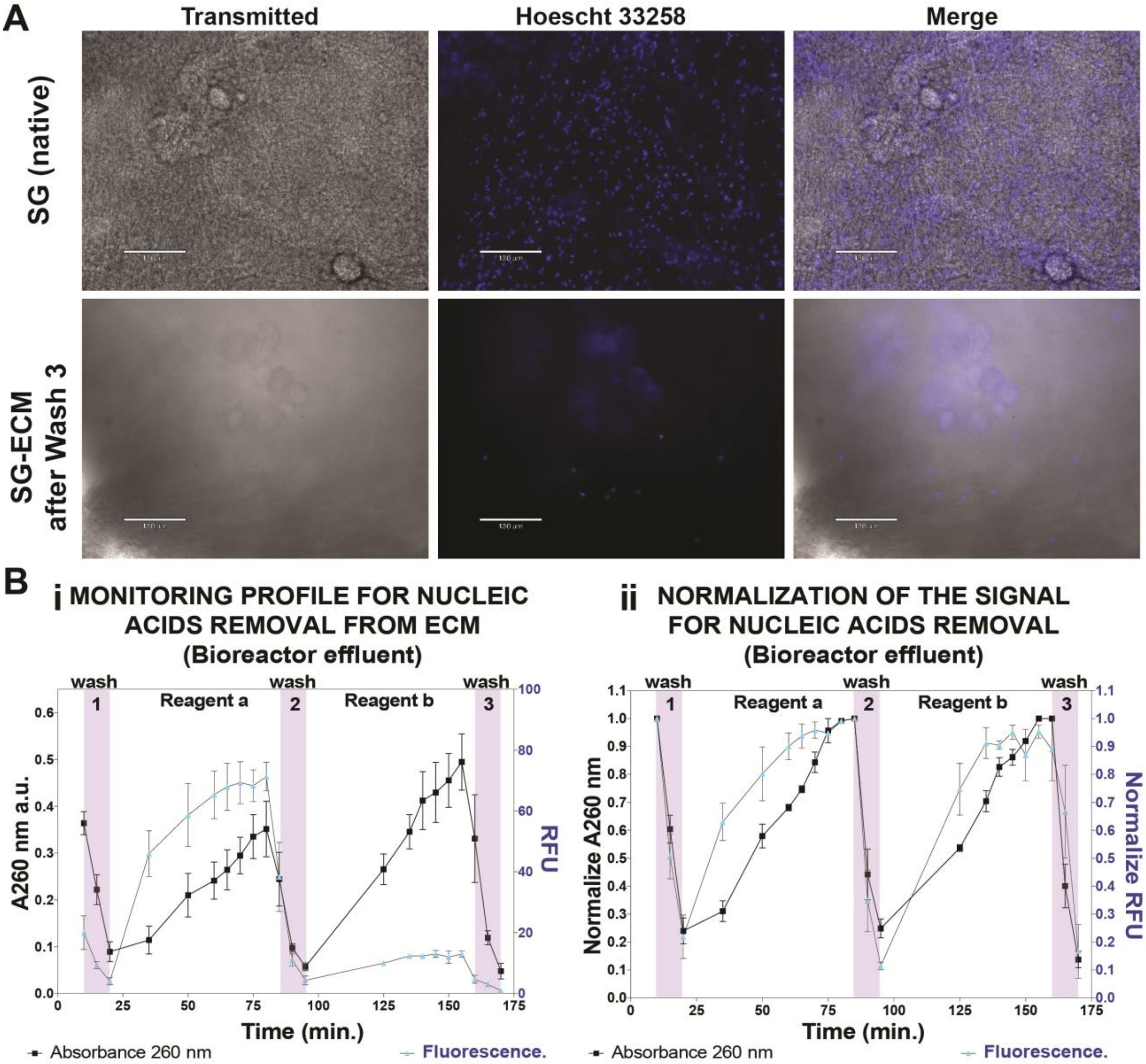

A) SG pre-stained with Hoechst 33258. The SG (native) raw shows representative images of the tissue before decellularization. SG-ECM raw shows the status of the tissue after wash 3 but without performing the Ster and final wash stage of the decellularization protocol as shown in the Table S1.

B) Absorbance and fluorescence curves tracing the decellularization status.

Figure S4. dsDNA titration under different chemistries

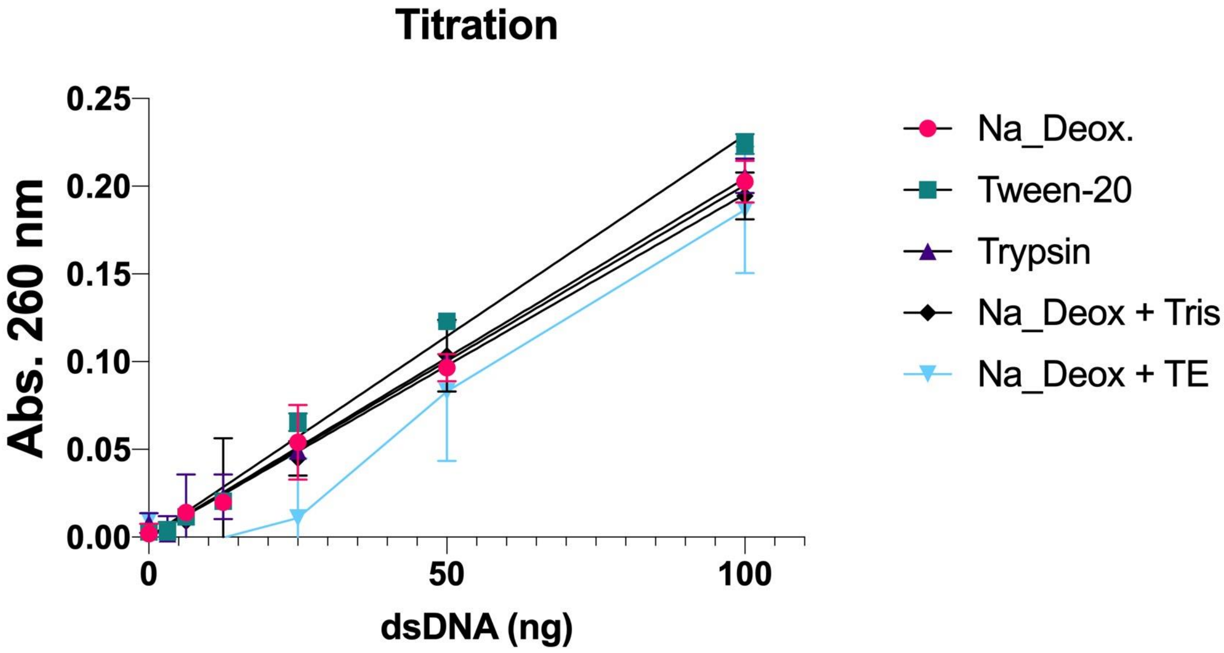

Absorbance values for standard lambda DNA diluted within different buffers and decellularization reagents (chemical environments). The error bars represent the SEM (n=3)

Figure S5. G' G" for hydrogel derived using manual decellularization method

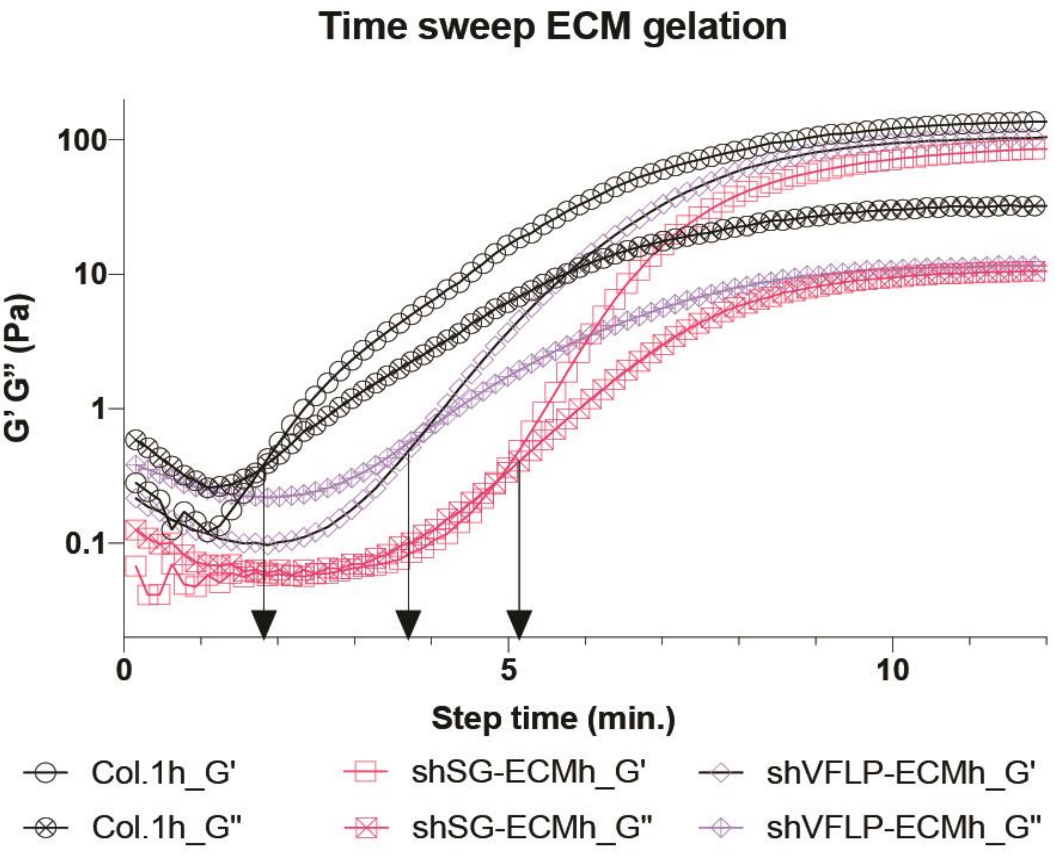

Figure S6. Log2 fold change (FC) auVFLP-ECM vs auSG-ECM. Proteins and subunits associated with ECM.

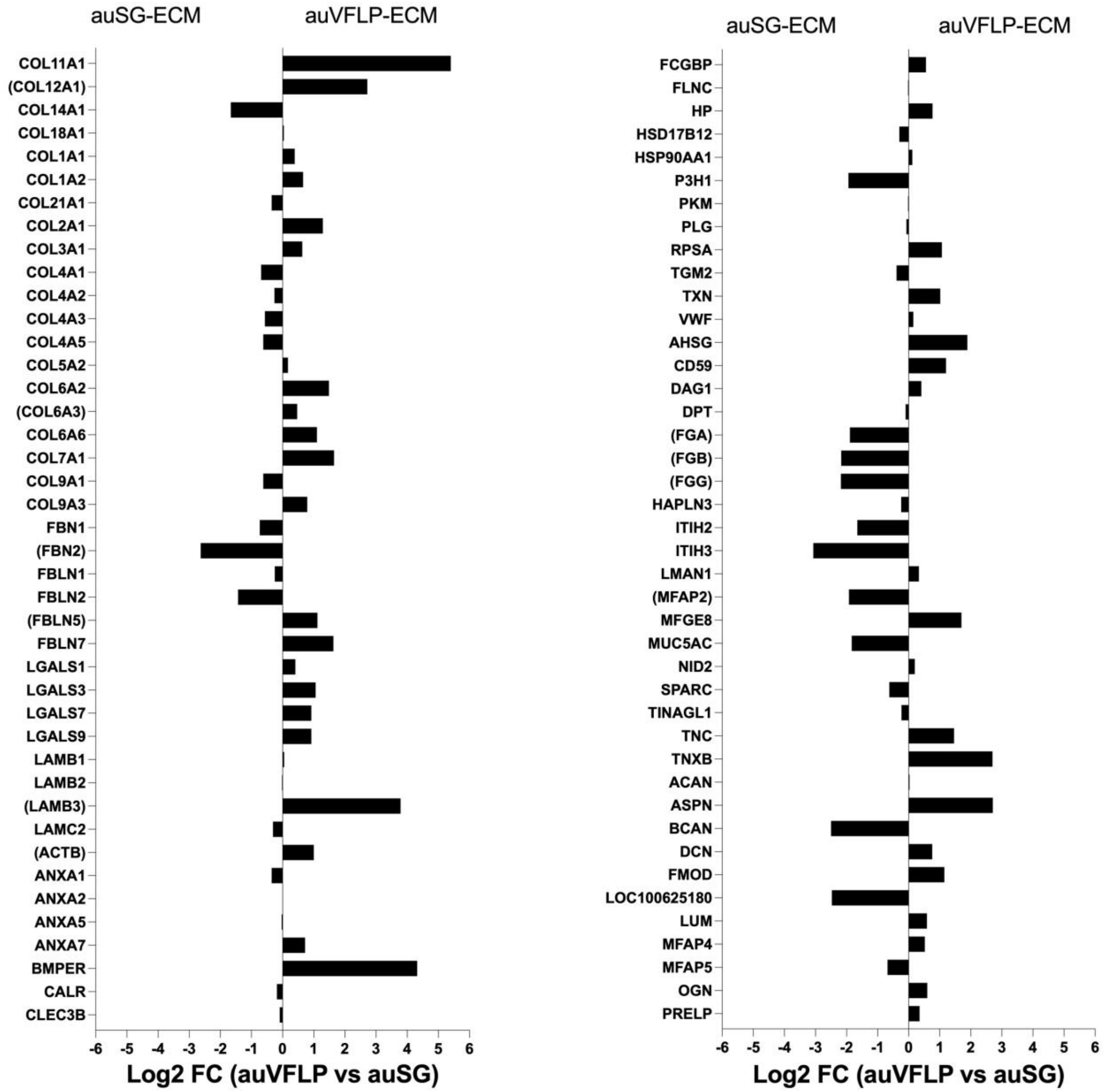

Figure S7.

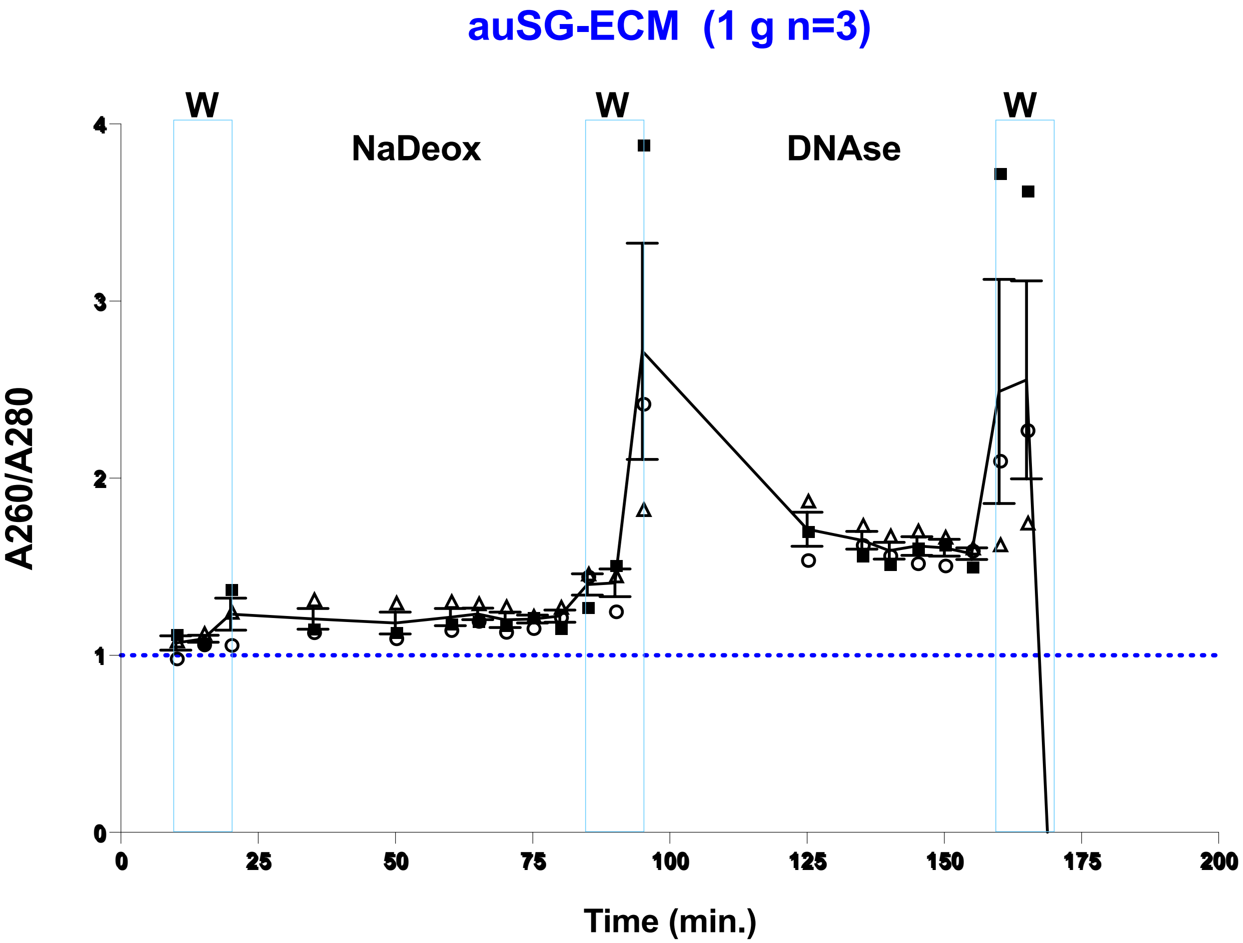

Abs260/Abs.280 ratio for Figure 3Bii au-SG-ECM. The drastic drop at the end was a signal interpreted as the wash has already finish and either Abs.260 or Abs.280 was acquired after measurement only noise absorbance.
